# Higher polygenic scores for C-reactive protein is associated with lower striatal reward magnitude coding in early adolescents

**DOI:** 10.1016/j.bbi.2026.106970

**Published:** 2026-08-23

**Authors:** Lily A. Heritage, Iris Ka-Yi Chat, Jonathan Ahern, Robert J. Loughnan, Robin L. Aupperle, Chun Chieh Fan, Martin P. Paulus, Wesley K. Thompson, Haixia Zheng

**Affiliations:** a Laureate Institute for Brain Research, Tulsa, OK, USA; b Department of Psychology, University of California, Los Angeles, USA; c Center for Population Neuroscience and Genetics, Tulsa, OK, USA; d Department of Cognitive Science, University of California, San Diego, La Jolla, CA, USA; e J. Craig Venter Institute, La Jolla, CA, USA; f Oxley College of Health and Natural Sciences, The University of Tulsa, Tulsa, OK, USA; g Department of Radiology, University of California School of Medicine, San Diego, CA, USA

**Keywords:** Inflammation, Polygenic score for C-reactive protein, fMRI Monetary incentive delay task, Reward anticipation, Striatum

## Abstract

Low-grade inflammation has been linked to altered reward processing and risk for mood disorders in adults, yet it remains unclear whether genetic liability for inflammation is associated with reward circuitry function before the typical age of onset for depression and anhedonia. Using data from the Adolescent Brain and Cognitive Development Study, we examined whether a polygenic score for C-reactive protein (PGS-CRP) was associated with striatal activation during reward processing in 4,830 children of European ancestry (ages 9–10). Participants com pleted the Monetary Incentive Delay task during functional MRI. Linear mixed-effects models tested associations between high versus low PGS-CRP and activation in the nucleus accumbens, caudate, and putamen during reward and loss anticipation and feedback, adjusting for sociodemographic, anthropometric, scanner, and genetic ancestry covariates. Higher PGS-CRP was associated with attenuated activation across all three striatal regions (i.e., the nucleus accumbens, caudate, and putamen) during anticipation of large relative to small rewards at baseline (all *p*_FDR_ < 0.05, beta coefficient range from −0.014 to −0.017). Associations were not observed for loss anticipation, average reward anticipation, or reward/loss feedback, suggesting altered reward-magnitude coding rather than generalized incentive hypoactivation. The caudate effect persisted across 6 years of longitudinal follow-up into mid-to-late adolescence and remained robust after adjustment for psychiatric diagnoses and psychotropic medication. These findings provide the earliest population-level evidence that genetic inflammatory liability is associated with attenuated striatal reward-value coding, identifying a candidate neuroimmune pathway preceding clinical risk.

## 1. Introduction

Adolescence is a developmental period marked by substantial changes in reward systems (Galván, 2010). These changes reflect coordinated development across multiple components of reward processing, including heightened responsivity to rewards and reward cues. These processes are supported by the maturation of corticostriatal circuit, including regions within the basal ganglia such as the nucleus accumbens, caudate, and putamen (Haber and Knutson, 2010). This maturation supports increasingly flexible, goal-directed behavior in complex environments (Telzer, 2016). However, substantial individual differences in reward-related brain function emerge during this period, and atypical development of these processes has been implicated in psychopathology, including depression and anhedonia (Nusslock and Alloy, 2017), as well as maladaptive health behaviors such as substance use (Casey and Jones, 2010) and sleep disruptions (Holm et al.,2009). Identifying biological mechanisms that contribute to variation in adolescent reward function is therefore critical for understanding early pathways of mental health risk.

Low-grade inflammatory signaling has gained increasing attention as one plausible mechanism shaping reward-related brain function. Peripheral inflammatory proteins, including interleukin-6 (IL-6), can access and modulate the brain through humoral, neural, and cellular pathways, including active transport, vagal signaling, and entry through circumventricular organs or more permeable blood–brain barrier (Dantzer et al., 2008; Dantzer, 2018). Circulating C-reactive protein (CRP), a hepatically synthesized acute-phase protein, is a downstream marker of inflammation produced in response to proinflammatory cytokines, particularly IL-6 (Pepys and Hirschfield, 2003). Although CRP remains sensitive to infection, adiposity, stress, and other physiological influences, its longer circulating half-life and greater analytic stability relative to many upstream cytokines make it a widely used downstream marker of low-grade systemic inflammation (Gabay and Kushner, 1999). Elevated CRP levels have been associated with altered reward-related brain function, including lower activation in the nucleus accumbens (Aruldass et al., 2025) and altered corticostriatal functional connectivity (Felger et al., 2016), particularly in individuals with depression. Experimental immune challenge studies using lipopolysaccharide (LPS) further support a mechanistic role, which demonstrates that greater LPS-induced IL-6 responses are associated with lower reward motivation at both behavioral (Chat et al., 2026a) and neural levels (Eisenberger et al., 2010). Elevated CRP has also been linked to greater increases in anhedonia (Savitz et al., 2025) following inflammatory challenge, and higher CRP levels have also been observed in a subset of individuals with depression (Osimo et al., 2019), a condition characterized by alterations in reward processing (Nestler and Carlezon, 2006). Together, these findings suggest that inflammatory activity may contribute to altered corticostriatal reward function, a pathway of particular relevance to depression and anhedonia.

Reward function can be dissociated into anticipatory and outcome-related components. This distinction is important because existing evidence suggests that inflammation may be especially relevant to anticipatory reward processes. Experimental research indicates that inflammatory activity is associated with reduced motivation, processes closely linked to reward anticipation (Chat et al., 2026b). These effects may reflect, in part, inflammation-associated alterations in dopaminergic signaling within the basal ganglia, which plays a central role in incentive motivation (Felger and Treadway, 2016). Consistent with this interpretation, studies that differentiate temporal components of anhedonia have reported stronger associations between inflammation and anticipatory, relative to consummatory, anhedonia (Chat et al., 2026b). This evidence suggests that inflammatory processes may contribute to variation in reward-related brain function, potentially with greater relevance for anticipatory mechanisms. At the same time, examining outcome-related or feedback-related responses can help determine whether inflammation-related associations are specific to anticipation or reflect broader alterations across different phases of reward processing.

These advances on inflammation and reward motivation focus on adults and it is unclear whether the link can be observed in adolescence, particularly in early adolescence, as empirical research remains limited. Existing studies in late adolescents suggest that the immune-reward associations are plausible. For example, CRP has been associated with altered resting-state corticostriatal functional connectivity (Liang et al., 2025), and inflammatory markers have been linked to altered activation in regions involved in reward processing (Bradley et al., 2019). However, a notable knowledge gap remains in early adolescence, a developmental period characterized by rapid maturation of reward circuitry, increasing environmental demands, and emerging individual differences in psychiatric risk. These factors may render reward systems particularly sensitive to biological influences, including immune processes, suggesting a potential window during which immune-related variation in reward function may be pronounced.

Despite growing interest in inflammation as a mechanism underlying reward-related brain function, studying circulating inflammatory markers in early adolescence presents several challenges. Widely used as a downstream marker of low-grade systemic inflammation, single-timepoint CRP levels are influenced by infection, adiposity, acute stress, and other transient physiological states (Sproston and Ashworth, 2018; Steptoe et al., 2007). These challenges may be amplified in early adolescence, when CRP levels are often low and variability may be restricted, complicating the detection of stable inflammation-related individual differences in neural function. Given these challenges, genetic approaches may provide a complementary strategy for studying inflammatory liability during this developmental period. A polygenic score for C-reactive protein (PGS-CRP) aggregates weighted CRP-associated genetic variants into a single index of an individual’s genetic propensity toward elevated circulating CRP levels, explaining approximately 16% of variance in plasma CRP levels (Said et al., 2022). Because PGS-CRP reflects a stable measurement that remains constant across the lifespan, its use as an aggregate genomic liability proxy for CRP reduces concerns regarding reverse causality and transient inflammatory fluctuations at the time of assessment. Additionally, because genomic data can be derived from minimally invasive sampling methods such as saliva collection, PGS-based approaches may be particularly well-suited to pediatric research contexts. Few studies have examined clinical implications of PGS-CRP in early adolescents. However, emerging evidence suggests that PGS-CRP is associated with maladaptive health behavior (Norton et al., 2025) and accelerated cortical thinning, both of which have been implicated in psychopathology (Zheng et al., 2026). This suggests that the health-related impacts of genetic liability for elevated CRP may emerge as early as early adolescence, yet no research has examined the association between PGS-CRP and reward-related brain function during this period.

To address this gap, the present study examined whether aggregate genomic liability for elevated CRP is associated with neural responses during reward processing in early adolescence. Given prior evidence linking inflammation most strongly to motivation and incentive processes, our primary focus was reward anticipation. Specifically, we tested associations between PGS-CRP and activation in corticostriatal reward regions, including the nucleus accumbens, caudate, and putamen, during the anticipation phase of a reward-processing task. We hypothesized that higher PGS-CRP would be associated with altered anticipatory reward-related activation in corticostriatal regions. To provide a fuller characterization of reward processing and assess whether associations were specific to anticipation, we also examined feedback-related neural responses as a complementary analysis. By integrating aggregate genomic liability for one measure of inflammation with neural measures of reward processing, this study aims to clarify an early-emerging biological pathway that may contribute to adolescent risk for depression, anhedonia, and related maladaptive health behaviors.

## 2. Methods

### 2.1. Participants and study procedures

The current study utilized data from the 6.1 release of the Adolescent Brain and Cognitive Development (ABCD) Study, an ongoing study following a baseline sample of 11,868 youth aged 9–10 (47.1% assigned female at birth) from 21 sites across the United States. Data collection began in September 2016 (Garavan et al., 2018) and the cutoff date for inclusion in this release was January 15, 2024 (ABCD Consortium, 2025).Consistent with ABCD Study^®^ protocols, families were compensated monetarily for their participation. Information about recruitment and sampling procedures for the ABCD Study has been described in detail elsewhere (Garavan et al., 2018).

Our primary analytical sample consists of cross-sectional data from the baseline visit (9–10 years old) and the three longitudinal follow-up visits at Years 2, 4, and 6. The sample was restricted to participants with genetically inferred European ancestry to better align with the European ancestry of the genome-wide association study (GWAS) discovery population (see section 2.3.; Ding et al., 2023). After excluding participants who: (1) lacked usable fMRI task data or did not meet fMRI quality control criteria (described in Casey et al., 2018); (2) had incomplete data for exposure, outcome, or covariate variables; or (3) did not meet criteria for genetically inferred European ancestry (see section 2.2.1), the analytical sample comprised N = 4,830 participants. Participant exclusions are shown in Fig. 1, and sample characteristics are summarized in Table 1 and Supplementary Table S1.

### 2.2. Measures

#### 2.2.1. Exposure variable: PGS-CRP

To examine how higher aggregate genomic liability for elevated CRP is associated with neural activation during reward anticipation, PGS-CRP was used as the primary exposure variable.

##### 2.2.1.1. Genome-wide association study.

PGS-CRP was generated using summary statistics drawn from a large-scale GWAS of CRP conducted in participants of European descent from the UK Biobank (N = 427,367) and the CHARGE Consortium (N = 575,531; Sollis et al., 2023), accessed via the NHGRI-EBI GWAS Catalog (accession GCST90029070; https://www.ebi.ac.uk/gwas/studies/GCST90029070). Summary statistics were harmonized and aligned to genome build GRCh38 using “cleansumstats” (Gadin et al., 2022).

##### 2.2.1.2. ABCD genetic data.

Genetic data were obtained from saliva samples collected as part of the ABCD Study (Uban et al., 2018). Genotyping was performed across 656,247 markers using the Smokescreen array (Baurley et al., 2016). To maximize variant overlap with the CRP GWAS, genotyped markers were imputed via the TOPMed imputation server (Taliun et al., 2021), yielding fractional dosages that were converted to integer allele counts using a best-guess threshold of 0.9, resulting in over 280 million imputed variants aligned to genome build GRCh38. Post-imputation, variants were restricted to autosomal SNPs with minor allele frequency ≥ 0.01, retaining approximately 11 million SNPs. Genetic continental ancestry was estimated using SNPweights (Chen et al., 2013) with reference panels representing African, East Asian, European (1000 Genomes Project Consortium et al., 2015) and Indigenous North and South American ancestry (Reich et al., 2012). Participants with inferred genetic ancestry at least 80% aligned with the European ancestry reference panels were categorized as of European ancestry (EUR ≥ 0.8).

##### 2.2.1.3. Polygenic score computation.

PGS-CRP was computed using PRScs (Ge et al., 2019), a Bayesian continuous shrinkage method selected based on prior benchmarking in the ABCD sample demonstrating maximized variance explained and cross-ancestry performance relative to pruning-and-thresholding approaches (Ahern et al., 2023). Posterior effect sizes were estimated using 10,000 MCMC iterations and 5,000 burn-in iterations; all other parameters were left at their defaults. Linkage disequilibrium reference panels were derived from 1000 Genomes Phase 3 and matched to the ancestry of the summary statistics. Posterior effect sizes were applied to ABCD target data to compute individual-level PGS values using PLINK 2.0 (Chang et al., 2015). PGS-CRP was dichotomized at the sample median for the primary analysis to compare participants with relatively higher versus lower genetic liability for elevated CRP. This grouping was used as an analytical contrast and does not imply a biological threshold at the median. The distribution of PGS-CRP and the median split are shown in Supplementary Fig. S1. Sensitivity analyses described in section 2.3.3. additionally modeled PGS-CRP as a continuous predictor and as quintiles of its distribution.

#### 2.2.2. Outcome variable: Neural activation in the MID task

##### 2.2.2.1. Task procedure.

The MID task was administered as part of the ABCD study (Casey et al., 2018), and separates anticipatory from feedback-related neural responses across varying incentive magnitudes, allowing us to index both sensitivity to reward magnitude and general incentive sensitivity. Participants underwent fMRI scanning while performing the MID task; neural responses were measured via Blood-Oxygen-Level-Dependent (BOLD) signals. There were five trial types that depended on the incentive cue shown at the start of each trial: (1) win $5.00 (large reward); (2) win $0.20 (small reward); (3) lose $0.20 (small loss); (4) lose $5.00 (large loss); and (5) no reward or loss (i.e., $0.00 at stake, neutral). Participants were presented one of the five cues for 2 s, which was then followed by a jittered anticipatory delay period of 1.5 to 4 s, and then a target stimulus that prompted participants to press a button in the scanner before the target disappeared. If participants succeeded at the trial, they either won the amount of money presented by the cue or avoided losing it. Target duration was adaptively adjusted to maintain approximately 60% accuracy. Outcome feedback indicating trial success (positive feedback) or failure (negative feedback) was displayed for 2 s. Each participant completed two task runs of approximately 5.5 min each, comprising 40 reward trials, 40 loss trials, and 20 neutral trials per run (Casey et al., 2018). The current study primarily uses fMRI data from the anticipatory phase of the MID task but also examines the data from the feedback phase in an exploratory analysis.

##### 2.2.2.2. fMRI data acquisition and analysis.

Neuroimaging data were collected every two years on the 3 T scanner platforms Siemens Prisma, GE MR750, and Philips Archieva dStream/Ingenia (Casey et al., 2018). Centralized preprocessing and first-level modeling were performed by the ABCD Data Analysis and Informatics Center (DAIC) using validated pipelines described in detail elsewhere (Casey et al., 2018; Hagler et al., 2019). Subcortical segmentation was carried out using FreeSurfer v5.3.0 (Fischl, 2012), and trial-level activation estimates were derived via a general linear model implemented in AFNI’s 3dDeconvolve (Cox, 1996), released as contrast-level beta weights representing BOLD percent signal change (PSC) between anticipation conditions, and between positive and negative feedback outcomes. Three striatal regions of interest were defined a priori: nucleus accumbens (NAc), caudate, and putamen. Activation estimates were averaged across the left and right hemispheres within each region, as our hypotheses concerned sensitivity to reward magnitude rather than hemispheric lateralization, and *meta*-analyses of the MID task report bilateral striatal engagement during reward anticipation without consistent lateralization (Oldham et al., 2018; Wilson et al., 2018). Bilateral averaging also improves the reliability of each regional estimate and reduces the number of models tested.

##### 2.2.2.3. Beta weight contrasts of interest.

For each contrast, beta coefficients were averaged across the two task runs. Two sets of anticipatory contrasts were used to index distinct aspects of incentive processing. To index magnitude sensitivity, four contrasts were used: (1) large reward (+$5.00) versus no incentive, (2) small reward (+$0.20) versus no incentive, (3) large loss (−$5.00) versus no incentive, and (4) small loss (−$0.20) versus no incentive. To index general incentive sensitivity, two contrasts were used: (1) reward anticipation (averaged across reward cue magnitudes: +$0.20 and +$5.00) versus no incentive and (2) loss anticipation (averaged across loss cue magnitudes: −$0.20 and −$5.00) versus no incentive. Temporal specificity was assessed utilizing two contrasts from the feedback phase of the MID task: (1) reward, positive vs. negative feedback and (2) loss, positive vs. negative feedback.

#### 2.2.3. Covariates

The fixed-effects covariates chosen for this study have been linked with systemic inflammation and mental health outcomes in existing literature (Peverill et al., 2021; Rakesh et al., 2022); these include age, assigned sex at birth, body mass index (BMI), self-reported race/ethnicity, caregiver education, household income, and the first ten principal components of genetic ancestry to account for population stratification (see section 2.3.; Fan et al., 2023). MRI manufacturer was additionally included as a fixed-effect covariate to account for scanner-induced systemic variance in BOLD signaling, independent of the substantive covariates above.

Three nested random intercepts were utilized to account for the nested structure of the ABCD Study data: observations were nested within participants (individual ID), participants within families (family ID), and families within the specific MRI scanners (MRI serial number). These intercepts account for within-participant correlation across ROIs, genetic and environmental similarity among siblings, and between-scanner variability in MRI acquisition.

### 2.3. Statistical analyses

Due to a phenomenon called population stratification, in which allele frequencies across ancestral groups reflect demographic history rather than trait-specific associations, PGS validity is reduced in samples whose ancestry differs from the GWAS discovery population (Duncan et al., 2019). The CRP GWAS used in the present study was conducted in participants of European descent, so to mitigate bias arising from population stratification and enhance the validity of the findings, we restricted our analytical sample to those with inferred European genetic ancestry (Ding et al., 2023). The ancestry inference procedure is described in section 2.2.1.

#### 2.3.1. Primary analyses

Striatal activation (in BOLD PSC) during reward anticipation was modeled using linear mixed-effects models (LME) implemented in the “nlme” package in R. For each contrast and ROI, the model took the form:

$$\gamma_{ijk=\beta_{0}+\beta_{1}(PGS-CRP_{i})+\beta_{2}(Condition_{ijk})+\beta_{3}(PGS-CRP_{i}\times Condition_{ijk})+X_{ijk}\beta +u_{i}+v_{j}+w_{k}+\varepsilon_{ijk}}$$

where *γ*_ijk_ is the activation beta weight (per contrast) for participant *i*, nested in family *j* and scanned on scanner *k*; *PGS* – *CRP_i_* is the median-split genetic liability group (low PGS-CRP group as reference group); *Condition_ijk_* is the within-person repeated-measures factor indexing which beta weight contrast was modeled for that observation, varying by model (described below). *X_ijk_* is a matrix of covariates including scanner manufacturer, age, sex, BMI, household income, caregiver education, and the first 10 principal components of genetic ancestry; *u_i_*, *v_j_*, and *w_k_* are random intercepts for MRI serial number, family ID, and individual ID, respectively. *ε_ijk_* represents the residual error. All models were run separately for each ROI (NAc, caudate, and putamen), and p-values for all effects were corrected for multiple tests using the Benjamini-Hochberg false discovery rate (FDR) procedure across the three ROIs within each effect.

The primary model (Model 1) tested if there were PGS-CRP group differences (high vs. low PGS-CRP) in striatal activation during reward anticipation across four conditions. The conditions had four levels corresponding to the magnitude sensitivity contrasts: small reward versus no incentive, large reward versus no incentive, small loss versus no incentive, and large loss versus no incentive. Small reward was the reference condition. The PGS-CRP x large reward interaction term is our primary effect of interest. Small and large loss conditions were retained in the same model to evaluate reward specificity.

This magnitude model was run cross-sectionally at baseline (Year 0), and longitudinally using available data from Years 0, 2, 4, and 6. The longitudinal model decomposed age into baseline age, capturing between-person differences at enrollment, and age change from baseline, capturing within-person developmental change. Sample sizes and mean ages at each imaging visit were as follows: Year 0 (N = 4,830, M = 9.97 years, SD = 0.63), Year 2 (N = 3,113, M = 12.0 years, SD = 0.65), Year 4 (N = 2,441, M = 14.2 years, SD = 0.73), and Year 6 (N = 1,772, M = 16.1 years, SD = 0.65).

#### 2.3.2. Specificity analyses

To determine whether the associations reflected generalized reward incentive hypoactivation or specific reward-magnitude coding, we examined average-reward and average-loss contrasts using the same statistical model (Model 2). To evaluate the temporal specificity, we utilized two contrasts from the feedback phase (i.e., reward feedback and loss feedback) instead of anticipation phase of the MID task (Model 3).

#### 2.3.3. Sensitivity analyses

A supplementary sensitivity analysis was conducted to evaluate whether the findings were robust to adjustment for psychiatric diagnoses and psychotropic medication use. Psychiatric diagnoses were obtained from the computerized version of the Kiddie Schedule for Affective Disorders and Schizophrenia (KSADS-COMP) caregiver report (Kaufman et al., 2021; Townsend et al., 2020). Psychotropic medication use reflects caregiver-reported medication in the two weeks preceding each visit and represents a derived variable based on medication categorization procedures described in prior ABCD literature (Lopez et al., 2026). Because Attention-Deficit/Hyperactivity Disorder (ADHD), Oppositional Defiant Disorder (ODD), Post-Traumatic Stress Disorder (PTSD), and other mood disorders (i.e., mood disorder diagnoses other than major depressive disorder, persistent depressive disorder, disruptive mood dysregulation disorder, and bipolar disorder) diagnoses were each significantly more prevalent in the high PGS-CRP group at baseline (see Table 1), these conditions were added as fixed-effect covariates in all cross-sectional models, alongside the covariates described in section 2.2.3. Current psychotropic medication use was also included as an additional fixed-effect covariate. In the longitudinal models, covariates were restricted to conditions assessed consistently across visits, thereby excluding ODD and other mood disorders from the longitudinal sensitivity analyses due to revisions to the KSADS-COMP assessment between visits. Time-varying covariates in the longitudinal sensitivity analyses were therefore ADHD, PTSD, and current psychotropic medication use. Models were fit to observations with complete data on these variables. Full psychiatric diagnosis and psychotropic medication data, including categorization criteria, are reported in Supplementary Tables S2 and S3, respectively.

To evaluate whether the primary findings depended on dichotomization of the exposure, Model 1 was refit with PGS-CRP entered as a continuous, z-scored predictor and, separately, as quintiles of the sample distribution with the lowest quintile as the reference group. All models retained the covariates, random-effects structure, and FDR correction used in the primary analysis and were run cross-sectionally and longitudinally.

Two additional sensitivity analyses evaluated whether pubertal maturation accounted for or moderated the longitudinal association. Perceived pubertal status was indexed by the continuous mean score of the caregiver-reported Pubertal Development Scale (PDS), administered at each imaging visit; caregiver report and the continuous mean score were selected over youth report and the categorical Tanner-stage approximation following published recommendations for the use of these data (Beltz et al., 2026; Cheng et al., 2021). The longitudinal Model 1 was refit with pubertal status included as a time-varying fixed-effect covariate and again including a three-way PGS-CRP x condition x pubertal status interaction. Both models retained the covariates, random-effects structure, and FDR correction used in the primary analysis.

## 3. Results

### 3.1. Sample characteristics

Demographic and clinical characteristics of the baseline analytical sample are presented in Table 1. PGS-CRP groups were well-matched on age and sex. However, several sociodemographic and clinical variables differed significantly between groups. Household income and caregiver education were modestly but significantly higher in the low PGS-CRP group (both *p* < 0.001), and the high PGS-CRP group included a slightly lower proportion of White participants (*p* = 0.03) and slightly higher mean BMI (*p* < 0.001). With respect to all psychiatric diagnoses (reported in Supplementary Table S2), four conditions were significantly more prevalent at baseline in the high PGS-CRP group relative to the low PGS-CRP group: ADHD (14.9% vs. 12.5%, *p* = 0.02), ODD (16.1% vs. 13.5%, *p* = 0.01), PTSD (1.9% vs. 1.0%, *p* = 0.01), and other mood disorders (6.7% vs. 4.2%, *p* < 0.001). In contrast, rates of major depressive disorder (MDD), bipolar disorder (BD), and generalized anxiety disorder (GAD) did not differ significantly between groups (all *p* ≥ 0.20) at baseline. Psychotropic medication use did not significantly differ between groups (*p* = 0.20). Rates of psychiatric diagnoses and psychotropic medication use over time can be found in Supplementary Tables S2 and S3.

### 3.2. Primary analyses: Reward-magnitude anticipation

#### 3.2.1. Model 1: PGS-CRP and striatal differentiation of large versus small reward anticipation

Higher PGS-CRP was associated with significantly lower striatal activation during anticipation of large relative to small rewards across all three ROIs after FDR correction (NAc: β = −0.017, 95% CI [−0.032, −0.002], *p*_FDR_ = 0.022; caudate: β = −0.017, 95% CI [−0.031, −0.004], *p*_FDR_ = 0.022; putamen: β = −0.014, 95% CI [−0.025, −0.002], *p*_FDR_ = 0.022). Thus, early adolescents with higher inflammatory genetic liability showed attenuated striatal differentiation of anticipated reward magnitude at baseline (Table 2; Fig. 2A).

The PGS-CRP x small or large loss anticipation interactions were not significant in any ROI (Table 2 and Fig. 2A). No main PGS-CRP effect was observed (Table 2). This pattern suggests specificity to reward-magnitude differentiation rather than broad alteration of motivational salience. Full model 1 results for all effects are available in Supplementary Table S4.

In the longitudinal magnitude model, the PGS-CRP x large reward interaction remained significant in the caudate (β = −0.013, 95% CI [−0.024, −0.003], *p*_FDR_ = 0.032) but did not survive FDR correction in the NAc (β = −0.011, 95% CI [−0.022, 0.001], *p*_FDR_ = 0.078) or putamen (β = −0.008, 95% CI [−0.016, 0.001], *p*_FDR_ = 0.078). Age-smoothed trajectories showed the most stable group separation in caudate, with greater overlap in NAc and putamen at later ages when sample sizes were smaller (Fig. 3; Supplementary Table S5; Supplementary Fig. S2A).

### 3.3. Specificity analyses

#### 3.3.1. Model 2: Average reward and loss anticipation

Average reward anticipation did not differ significantly by PGS-CRP group in any ROI after FDR correction (all *p*_FDR_ ≥ 0.440; Supplementary Table S6; Fig. 2B), and PGS-CRP x average loss interactions were also non-significant (all *p*_FDR_ ≥ 0.434; Supplementary Table S6; Fig. 2B). These null average-contrast findings support the interpretation that the primary association reflects altered coding of reward magnitude rather than generalized reward hypoactivation. Longitudinal average reward/loss models were likewise non-significant across ROIs (Supplementary Table S7; Supplementary Fig. S2B).

#### 3.3.2. Model 3: Reward and loss feedback phase

PGS-CRP was not significantly associated with reward or loss feedback responses in any striatal ROI, cross-sectionally or longitudinally (Supplementary Tables S8–S10). These findings suggest that the observed association is more evident during anticipatory valuation than during outcome feedback.

### 3.4. Sensitivity analyses

#### 3.4.1. Clinical covariates

In the cross-sectional models, the significant PGS-CRP x large reward anticipation interaction across all three striatal ROIs was preserved, as were the null effects for all other anticipation contrasts and for the secondary feedback model, after adjusting for ADHD, ODD, other mood disorders, and PTSD diagnoses, and current psychotropic medication use (Supplementary Tables S11–S13).

In the longitudinal models, the PGS-CRP x large reward anticipation interaction remained significant in the caudate after FDR correction after adjustment for ADHD and PTSD diagnoses, as well as current psychotropic medication use. The null effects for Models 2 and 3 persisted as well (Supplementary Tables S14–S16).

#### 3.4.2. Continuous and quintile PGS-CRP

Sensitivity analyses treating PGS-CRP as a continuous predictor showed PGS-CRP x large reward interaction associations in the same direction as the primary analysis, although these effects did not survive FDR correction cross-sectionally or longitudinally (Supplementary Table S17). Quintile analyses did not indicate a clear monotonic association across the PGS-CRP distribution (Supplementary Table S18; Supplementary Fig. S3).

#### 3.4.3. Pubertal status

Perceived pubertal status advanced substantially across the four imaging visits in both sexes (Supplementary Table S19, Supplementary Fig. S4) and was strongly correlated with within-person change in age (r = 0.79). Pubertal variability at baseline was minimal, particularly among male participants (PDS mean = 1.36, SD = 0.30), indicating that the primary cross-sectional association at ages 9–10 is unlikely to reflect pubertal differences. Including time-varying pubertal status as an additional fixed-effect covariate left the longitudinal findings unchanged, with the PGS-CRP x large reward anticipation interaction remaining significant in the caudate after FDR correction (Supplementary Table S20). Adding a three-way PGS-CRP x condition x pubertal status interaction likewise left the primary interaction unchanged, and no three-way term was significant in any ROI (Supplementary Table S21). These findings suggest that the observed association was neither accounted for by nor moderated by pubertal maturation across follow-up.

## 4. Discussion

The current study observed higher PGS-CRP was associated with attenuated activation across nucleus accumbens, caudate, and putamen during anticipation of large relative to small rewards — a pattern that was specific to reward anticipation, was not observed for loss anticipation or reward feedback, and was sustained in the caudate area across longitudinal follow-up into mid-adolescence. Critically, this association was not driven by global hypoactivation to incentive cues but reflected a narrowing of the neural gradient encoding reward magnitude, suggesting that a higher aggregate genomic predisposition toward elevated CRP may impair the precision of incentive valuation (reward magnitude coding) rather than global reward detection. Together, these results provide the earliest population-level evidence that aggregated genomic propensity toward elevated CRP is associated with the variation of reward circuit function at a developmental stage preceding the peak-risk window for depression and anhedonia.

The selectivity of the PGS-CRP association (i.e., confined to reward magnitude discrimination during anticipation, absent during loss anticipation and reward feedback) is mechanistically informative and consistent with the known neurobiological targets of inflammatory signaling. Proinflammatory cytokines, particularly IL-6 and TNF-α, reduce striatal dopamine synthesis and release partly through activation of indoleamine 2,3-dioxygenase (IDO), degrading the precision of dopaminergic reward prediction signals rather than abolishing incentive responsivity altogether (Felger and Treadway, 2016; Treadway et al., 2019). This mechanistic account predicts precisely the pattern observed here: intact general incentive detection reflected in the null PGS-CRP main effect and overall reward cue in Model 2, alongside impaired magnitude-dependent scaling (i.e., the significant PGS-CRP × large reward interaction in Model 1) because the latter depends on phasic dopaminergic gradient coding whereas the former can be sustained by partially preserved tonic signaling. The temporal specificity of the association, present during anticipation but absent during feedback, further localizes the deficit to incentive-motivational rather than hedonic or evaluative processes. This is consistent with the theoretical distinction between dopaminergically-mediated reward ‘wanting’ and opioidergically-mediated reward ‘liking’ (Berridge and Robinson, 2003), and with experimental evidence that inflammatory challenge preferentially reduces anticipatory motivation and approach behavior while leaving consummatory pleasure comparatively intact (Eisenberger et al., 2010). Notably, some of the existing literature examining inflammation and reward-related brain function has focused on broad contrasts between reward versus no-reward conditions (Eisenberger et al., 2010; Liu et al., 2020), which would be insensitive to the more nuanced impairment in within-valence magnitude encoding documented here. More recent experimental inflammatory challenge studies via endotoxin and LPS administration suggested reduced reward sensitivity preferentially under conditions requiring discrimination between reward magnitudes rather than producing uniform suppression of reward-cue responsivity (Boyle et al., 2025; Chat et al., 2026a). Our findings therefore offer a potential resolution for prior null or weak results in this literature: inflammation-related effects on striatal function may only be detectable when the neural contrast is designed to capture gradient coding of incentive value during reward anticipation rather than binary reward detection.

The longitudinal trajectories reveal a regionally differentiated pattern that carries developmental signal beyond what the statistical model estimates convey. As shown in Fig. 3, in the nucleus accumbens, the high versus low PGS-CRP group difference in large reward anticipation activation was present at ages 9–10 but attenuated by early-to-mid adolescence (~age 13), suggesting that the NAc association may be concentrated in the prepubertal window when mesolimbic dopamine receptor density is near its developmental peak (Larsen et al., 2020) and the NAc is undergoing particularly rapid synaptic remodeling. By contrast, the caudate showed a sustained high versus low group difference across the full age range observed, with the low PGS-CRP group exhibiting a steeper activation trajectory through mid-to-late adolescence. This association is consistent with the caudate’s comparatively protracted maturation of fronto-striatal connectivity relative to the NAc (Insel et al., 2017), potentially maintaining sensitivity to inflammatory genetic input over a longer developmental window. The putamen trajectory was less interpretable given substantial confidence interval overlap beyond age 16, likely reflecting smaller within-person observation counts at later timepoints. Together, these trajectories suggest that the neural consequences of inflammatory genetic liability on reward coding are not uniformly attenuating with development, but rather regionally specific: transient in the NAc, persistent in the caudate. The pattern of more robust cross-sectional than longitudinal associations is consistent with this interpretation and may reflect the increasing moderating influence of experiential and contextual factors as adolescents accumulate broader environmental exposures, which would progressively attenuate the relative contribution of genomic liability to reward-related neural function (Lenroot and Giedd, 2011; van Dongen et al., 2016). Alternatively, neuroimmune influences on reward-related brain function may be particularly salient during earlier developmental periods when reward circuits are undergoing rapid maturation (Brenhouse and Schwarz, 2016). Pubertal status neither accounted for nor moderated the longitudinal association (see section 3.4.3), indicating that the regional differences in developmental course are unlikely to reflect differences in pubertal maturation. These interpretations are provisional; establishing whether this attenuation represents true developmental moderation or statistical artifact requires larger longitudinal samples with richer environmental and hormonal characterization, as well as replication cohorts spanning preadolescence through young adulthood.

The clinical significance of the observed PGS-CRP effect on striatal reward magnitude discrimination remains to be established, but several patterns in Table 1 offer preliminary context. The elevated rates of ADHD and ODD in the high PGS-CRP group are consistent with our prior report in the same ABCD cohort demonstrating that higher PGS-CRP is associated with increased externalizing psychopathology (Zheng et al., 2026), though these differences did not persist beyond the baseline visit (Supplementary Table S2). Both disorders are characterized by reduced striatal activation during MID reward anticipation in children (Scheres et al., 2007), a neural profile that closely mirrors the PGS-CRP associated pattern observed in the current study. Importantly, the PGS-CRP effect on striatal reward magnitude remained robust after covarying for psychiatric diagnoses and psychotropic medication use in the sensitivity analysis, indicating that the observed neural association is not attributable to between-group diagnostic differences and may instead reflect a shared neuroimmune substrate operating upstream of clinical diagnostic threshold. The absence of MDD group differences likely reflects the low base rate in this age group (2.3%) rather than a true null effect. MDD prevalence in this sample rose to 8.4% by Year 6 and psychotropic medication use from 9.3% to 23.6% (Supplementary Tables S2 and S3; stratified by PGS-CRP group), indicating that the cohort is entering the developmental window in which depressive disorders emerge. The neural association reported here therefore precedes rather than coincides with the period of peak risk, although whether it forecasts subsequent onset remains untested. This further supports that the enrichment of subthreshold mood disorders without differences in fully diagnosable affective syndromes may reflect the early developmental stage of the baseline sample, though this pattern requires prospective follow-up to interpret. The elevated PTSD prevalence in the high PGS-CRP group is intriguing but, given the low overall rate (1.4%), should be treated as hypothesis-generating. Whether the neural differences documented here confer risk for later psychopathology remains an open empirical question requiring longitudinal follow-up linking PGS-CRP, striatal reward function, and psychiatric outcomes across development.

This study leveraged a large longitudinal developmental dataset, allowing examination of the associations between PGS-CRP and reward-related brain function during early adolescence. The use of an aggregate genomic liability proxy for CRP reduced concerns regarding reverse causality and transient inflammatory fluctuations at the time of assessment. The integration of genomic and fMRI data within an early adolescent sample addresses an under-examined developmental period that may provide critical insight into when inflammation-related processes begin to shape reward circuitry. The findings should also be interpreted considering several limitations. First, and most consequentially, circulating CRP and other inflammatory biomarkers were not available in the ABCD baseline release used here, preventing mediation analysis that would establish whether PGS-CRP effects on striatal function are indeed transduced through elevated CRP protein levels or through correlated genetic pathways. PGS-CRP explains approximately 16% of variance in plasma CRP (Said et al., 2022), meaning the majority of CRP variance and potentially distinct inflammatory mechanisms and environmental interactions are not fully captured; the neural association reported here could reflect pleiotropic genetic effects on immune and neural development that are not fully reducible to CRP biology. Future work in samples with concurrent proteomics will be essential to establish the specific inflammatory pathway. Second, early adolescence is a developmentally sensitive period characterized by sex-dependent changes in both immune and reward-related processes (Jarkas et al., 2024; Lasselin et al., 2018). Nonetheless, because analyses involving polygenic scores require large sample sizes, the present study was under-powered to robustly examine sex-specific associations. Although perceived pubertal status neither accounted for nor moderated the longitudinal association (section 3.4.3), the PDS reflects perceived rather than clinically assessed maturation, and circulating reproductive hormone data were not incorporated here. More precise characterization of pubertal and hormonal contributions, including sex-specific effects, will require larger samples with direct hormonal assessment. Third, although restriction to participants of genetically inferred European ancestry was necessary to maintain validity of UK Biobank GWAS data to derive PGS-CRP, it limits generalizability of findings (Ding et al., 2023) and highlights the need for further investigation using a more ancestrally diverse GWAS discovery population. Fourth, the primary median-split analysis should be interpreted as a comparison between relatively higher and lower genetic liability rather than evidence for a biological threshold. Continuous and quintile sensitivity analyses produced directionally consistent but more heterogenous results and did not establish a specific dose–response relationship. Finally, although the MID task is well-validated, monetary reward may not represent the most developmentally salient incentive for all youth. Future research should investigate whether similar associations emerge across other forms of reward such as social reward tasks or ecological momentary paradigms, especially those more relevant to this developmental period (e.g., play time, positive social interactions).

### 4.1. Conclusion

Together, this study provides population-level evidence that aggregate genomic liability toward elevated systemic inflammation, represented by CRP, is associated with attenuated striatal differentiation of anticipated reward magnitude in early adolescence. Rather than a generalized reduction in incentive responsivity, we observed a pattern that was specific to reward magnitude coding during anticipation, was absent during loss anticipation and feedback phase, and remained robust after accounting for psychiatric diagnoses and psychotropic medication use. These findings extend neuroimmune models of psychiatric risk by indicating that inflammation-related genetic liability is linked to reward-circuit function before the typical peak period for depression and anhedonia onset. Whether this early narrowing of reward-magnitude coding constitutes a measurable step on the path to depression, anhedonia, or externalizing psychopathology is the question these data raise but cannot resolve. Future work integrating polygenic liability, circulating inflammatory markers, and prospective psychiatric outcomes will be essential for determining whether altered striatal reward-value coding represents a mechanism through which inflammatory biology contributes to later motivational and affective psychopathology.

## Supplementary Material

supplementary material

## Figures and Tables

**Fig. 1.**
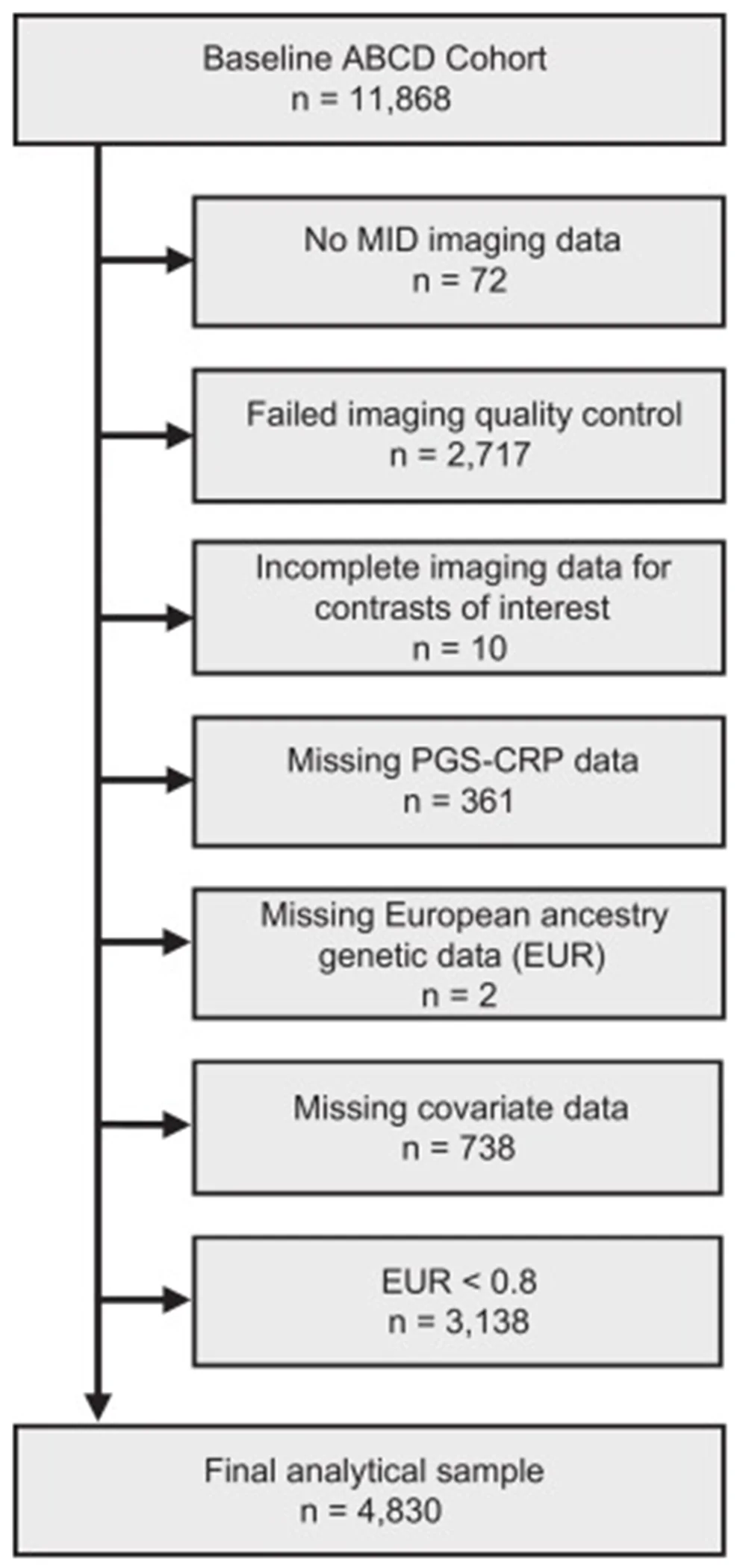
Flow diagram of participant selection *Note*. Participant selection flowchart for the final analytical sample. The longitudinal sensitivity analysis uses the same analytical sample as the cross-sectional primary analysis. *Abbreviations:* ABCD = Adolescent Behavioral Cognitive Development Study; MID = Monetary Incentive Delay task; PGS-CRP = polygenic score for C-reactive protein; EUR < 0.8 = European genetic continental ancestry proportion below 0.80.

**Fig. 2.**
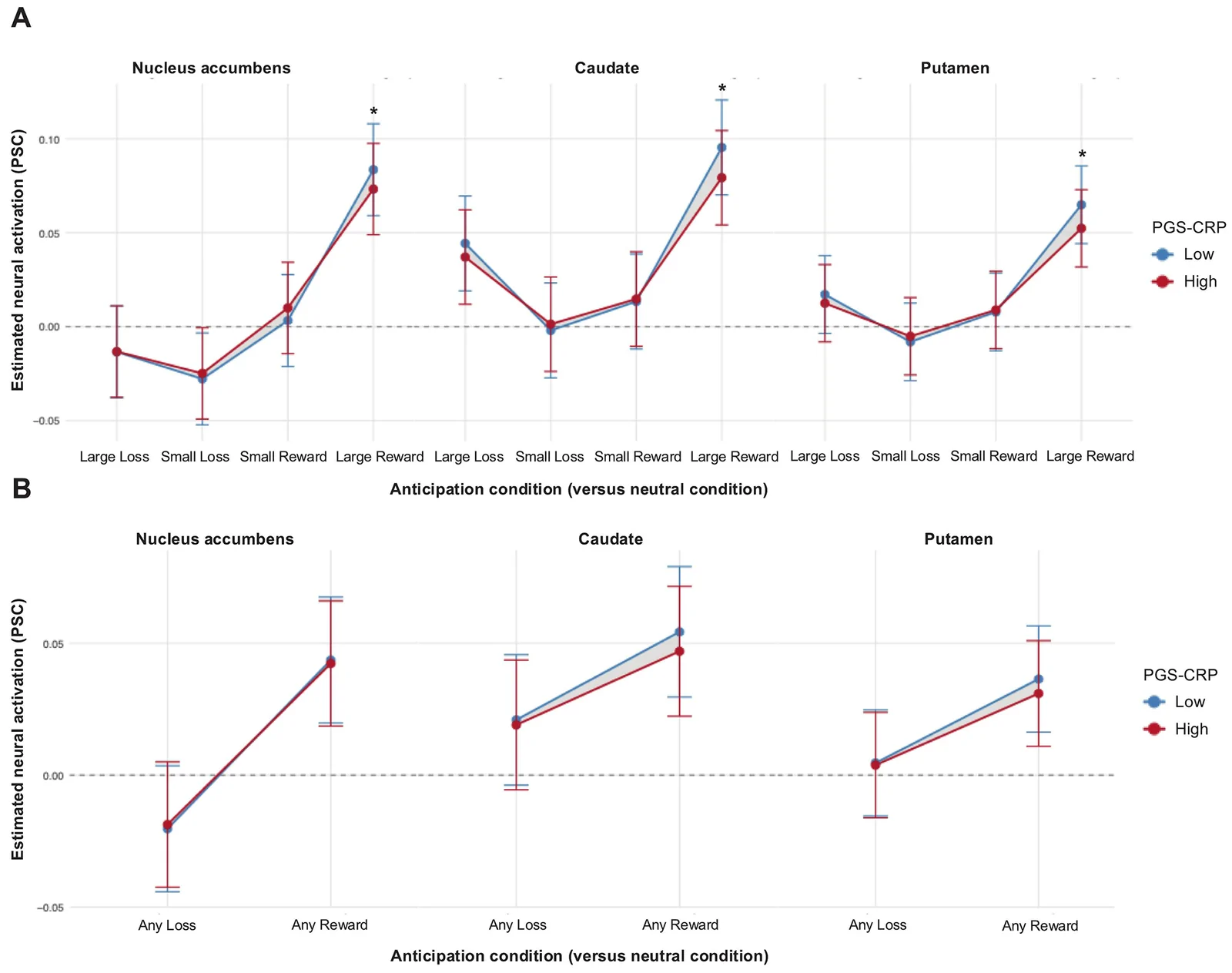
PGS-CRP group differences in striatal reward-magnitude anticipation. *Note*. Estimated marginal means (EMMs) for striatal activation during anticipation conditions of the Monetary Incentive Delay (MID) task, relative to the neutral anticipation condition, for High and Low PGS-CRP groups in the nucleus accumbens, caudate, and putamen. Error bars represent 95% confidence intervals. Asterisks denote False Discovery Rate (FDR)-corrected significant effects (*p*_FDR_ <0.05). (A) Model 1: EMMs across four anticipation conditions (large loss = −$5.00, small loss = −$0.20, small reward = +$0.20, large reward = +$5.00). A significant PGS-CRP × large reward interaction was observed at large reward anticipation in all three ROIs, with the low PGS-CRP group showing greater activation than the high PGS-CRP group. (B) Model 2: EMMs for any reward (mean activation to +$0.20 and +$5.00 cues vs. neutral cue) and any loss (mean activation to −$0.20 and −$5.00 cues vs. neutral cue) anticipation conditions. No significant group differences were observed. *Abbreviations:* PSC = percent blood oxygen level (BOLD) dependent signal change between anticipatory condition vs. neutral cue; PGS-CRP = polygenic score for C-reactive protein.

**Fig. 3.**
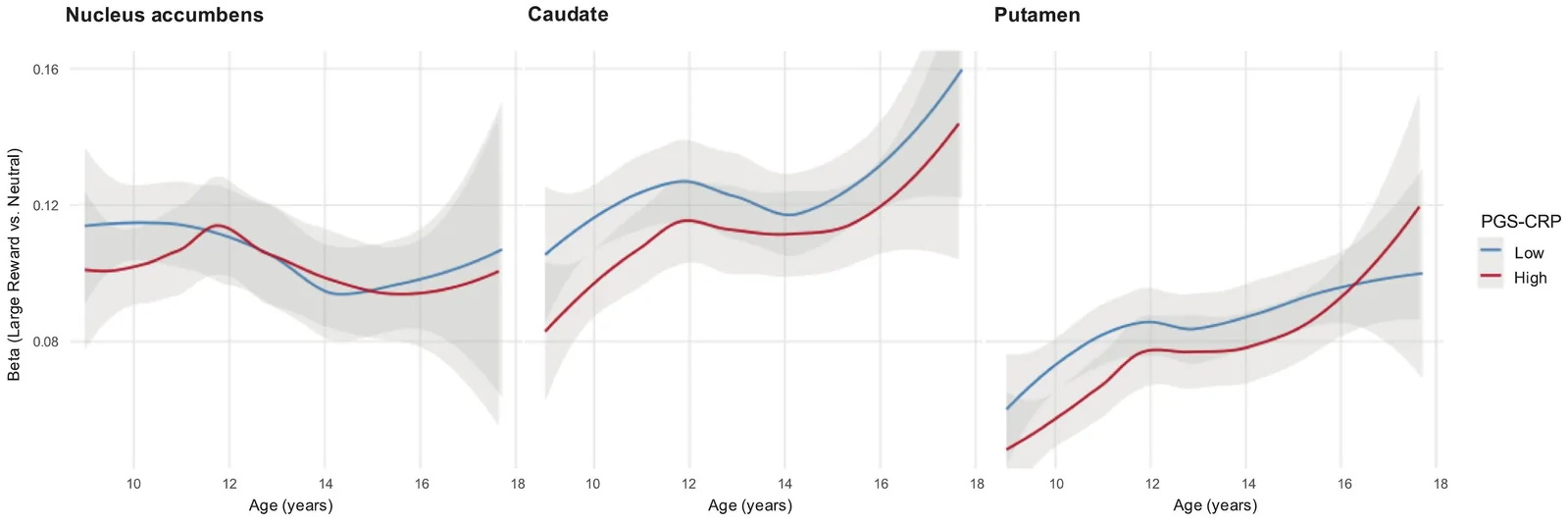
Longitudinal trajectories of striatal activation during large reward anticipation. *Note*. Plots show locally smoothed average beta values for the large reward vs. neutral Monetary Incentive Delay (MID) contrast by age, unadjusted for covariates. Shaded regions indicate 95% confidence intervals (CI). Wide CIs at age extremes reflect smaller sample sizes at those timepoints. *Abbreviations:* PGS-CRP = polygenic score for C-reactive protein.

**Table 1:** Demographic characteristics of analytical sample by PGS-CRP group.

Variable	OverallN = 4,830	Low PGS-CRPN = 2,408	High PGS-CRPN = 2,422	*p*
Age, years [M(SD)]	9.97 (0.63)	9.98 (0.63)	9.96 (0.62)	0.30
Sex, female [n(%)]	2,329 (48.2%)	1,155 (48.0%)	1,174 (48.5%)	0.70
BMI [M(SD)]	17.87 (3.25)	17.62 (3.00)	18.12 (3.46)	<0.001
Household income, $100 k–200 k [n(%)]	1,985 (41.1%)	1,024 (42.5%)	961 (39.7%)	<0.001
Caregiver education, graduate/professional [n(%)]	2,248 (46.5%)	1,173 (48.7%)	1,075 (44.4%)	<0.001
Race/ethnicity, White [n(%)]	4,452 (92.2%)	2,244 (93.2%)	2,208 (91.2%)	0.03
Psychiatric diagnoses [n(%)]				
*ADHD*	661 (13.7%)	300 (12.5%)	361 (14.9%)	0.02
*ODD*	717 (14.8%)	326 (13.5%)	391 (16.1%)	0.01
*MDD*	111 (2.3%)	51 (2.1%)	60 (2.5%)	0.50
*BD* ^a^	104 (2.2%)	49 (2.0%)	55 (2.3%)	0.60
*PDD*	6 (0.1%)	3 (0.1%)	3 (0.1%)	>0.9
*DMDD*	4 (0.1%)	3 (0.1%)	1 (0.0%)	0.6
*Other mood disorders* ^b^	264 (5.5%)	102 (4.2%)	162 (6.7%)	<0.001
*Schizophrenia*	0 (0.0%)	0 (0.0%)	0 (0.0%)	
*PTSD*	68 (1.4%)	23 (1.0%)	45 (1.9%)	0.01
*GAD*	243 (5.0%)	116 (4.8%)	127 (5.2%)	0.50
Any psychotropic medication [n(%)]	448 (9.3%)	209 (8.7%)	239 (9.9%)	0.20

*Note*. Group differences were assessed using Welch’s two-sample *t*-test (continuous variables) or Pearson’s chi-squared test (categorical variables). The analytical sample was restricted to participants of genetically inferred European ancestry to align with the UK Biobank GWAS discovery population. Psychiatric diagnoses were obtained from caregiver report via the Kiddie Schedule for Affective Disorders and Schizophrenia for DSM-5 (KSADS-5). Any psychotropic medication use reflects caregiver-reported medication in the two weeks prior to the baseline visit and represents a derived variable based on medication categorization procedures described in prior ABCD literature (Lopez et al., 2026). Full psychiatric diagnosis and psychotropic medication data, including categorization criteria, are reported in Supplementary Tables S2 and S3, respectively.

*Abbreviations:* PGS-CRP = polygenic score for C-reactive protein; M = mean; SD = standard deviation; n = frequency; % = percent; BMI = body mass index; $100 k-$200 k = $100,000 to $200,000 USD; ADHD = attention-deficit/hyperactivity disorder; ODD = oppositional defiant disorder; MDD = major depressive disorder; PDD = persistent depressive disorder; DMDD = disruptive mood dysregulation disorder; BD = bipolar disorder; PTSD = post-traumatic stress disorder; GAD = generalized anxiety disorder.

a BD includes bipolar I and II diagnoses

b Other mood disorders include specified depressive disorders other than MDD, PDD, and DMDD, and specified and unspecified bipolar disorders other than bipolar I and II (i.e., subthreshold or minimum duration).

**Table 2:** Association between PGS-CRP and striatal sensitivity to reward anticipation magnitudes.

ROI	β	95% CI	*p*	*p*_FDR_
**PGS-CRP Main Effect on Small Reward Anticipation** ^a^				
NAc	0.007	[−0.008, 0.022]	0.373	0.862
Caudate	0.001	[−0.013, 0.015]	0.853	0.862
Putamen	0.001	[−0.011, 0.013]	0.862	0.862
**PGS-CRP x Large Reward Anticipation Interaction**				
NAc	−0.017	[−0.032, −0.002]	0.022*	0.022*
Caudate	−0.017	[−0.031, −0.004]	0.013*	0.022*
Putamen	−0.014	[−0.025, −0.002]	0.021*	0.022*
**PGS-CRP x Small Loss Anticipation Interaction**				
NAc	−0.004	[−0.018, 0.011]	0.610	0.779
Caudate	0.002	[−0.012, 0.016]	0.779	0.779
Putamen	0.002	[−0.010, 0.014]	0.743	0.779
**PGS-CRP x Large Loss Anticipation Interaction**				
NAc	−0.007	[−0.021, 0.008]	0.381	0.381
Caudate	−0.009	[−0.022, 0.005]	0.223	0.381
Putamen	−0.006	[−0.017, 0.006]	0.334	0.381

*Note*. The PGS-CRP main effect estimates the difference in striatal activation between High and Low PGS-CRP groups during small reward anticipation. Each interaction term estimates the additional shift in this group difference when the condition is large reward, small loss, or large loss anticipation compared to small reward anticipation. Significant negative PGS-CRP x large reward anticipation interactions were observed across all three ROIs (all *p*_FDR_ = 0.022), indicating reduced striatal activation in the High relative to Low PGS-CRP group during large reward anticipation compared to small reward anticipation. PGS-CRP x small loss and large loss anticipation interactions were non-significant across all ROIs (all *p*_FDR_ > 0.77).

*Abbreviations:* ROI = region of interest; β = beta coefficient representing the difference in Blood Oxygen Level Dependent percent signal change between High and Low PGS-CRP groups; *p*_FDR_ = Benjamini-Hochberg False Discovery Rate corrected p-value; PGS-CRP = polygenic score for C-reactive protein; NAc = nucleus accumbens.

a The small reward vs. neutral contrast serves as the reference condition in Model 1. The main effect of PGS-CRP in the model is on the small reward anticipation condition.

## Data Availability

Data are from the ABCD Study, curated release 6.1, available via the NIMH Data Archive (NDA) ABCD collection (https://nda.nih.gov/abcd). Access requires NDA Data Use Certification. This study used data from the Adolescent Brain Cognitive Development (ABCD) Study, curated release 6.1. These data are publicly available through the National Institute of Mental Health Data Archive (NDA) under the ABCD collection (https://nda.nih.gov/abcd). Access requires completion of the NDA Data Use Certification.

## Appendix A. Supplementary data

Supplementary data to this article can be found online at https://doi.org/10.1016/j.bbi.2026.106970.
