# Supplementary material for: Higher polygenic scores for C-reactive protein is associated with lower striatal reward magnitude coding in early adolescents
Source: Brain Behav Immun. Author manuscript; Available in PMC 2026 Oct 22. (PMC13690263; doi:10.1016/j.bbi.2026.106970)
Supplement: Supplementary file 1 [file NIHMS2213047-supplement-supplementary_material.docx]

Table of Contents

[Supplementary Table S1. Baseline Characteristics of Participants Contributing Data at Each Imaging Visit by PGS-CRP Group	3](#_Toc238208068)

[Supplementary Figure S1. Distribution of PGS-CRP Scores in the Baseline Analytical Sample	5](#_Toc238208069)

[Supplementary Table S2. Caregiver-Reported Psychiatric Diagnoses by Imaging Visit and PGS-CRP Group	6](#_Toc238208070)

[Supplementary Table S3. Caregiver-Reported Psychotropic Medication Use by Imaging Visit and PGS-CRP Group	8](#_Toc238208071)

[Supplementary Table S4. Complete Fixed-Effect Output for Cross-Sectional Model 1	9](#_Toc238208072)

[Supplementary Table S5. Summary of Model 1 Longitudinal Sensitivity Analysis Effects	14](#_Toc238208073)

[Supplementary Figure S2. Longitudinal Sensitivity Analysis (Models 1 and 2): PGS-CRP Group Differences in Striatal Activation During Reward and Loss Anticipation in the MID Task	16](#_Toc238208074)

[Supplementary Table S6. Complete Fixed-Effect Output for Cross-Sectional Model 2	17](#_Toc238208075)

[Supplementary Table S7. Summary of Model 2 Longitudinal Sensitivity Analysis Effects	23](#_Toc238208076)

[Supplementary Table S8. PGS-CRP Effect Estimates for Striatal Activation During Reward and Loss Feedback (Cross-Sectional Model 3)	24](#_Toc238208077)

[Supplementary Table S9. Complete Fixed-Effect Output for Cross-Sectional Model 3	25](#_Toc238208078)

[Supplementary Table S10. Summary of Model 3 Longitudinal Sensitivity Analysis Effects	30](#_Toc238208079)

[Supplementary Table S11. Summary of Cross-Sectional Model 1 Clinical Covariate Sensitivity Analysis Effects	31](#_Toc238208080)

[Supplementary Table S12. Summary of Cross-Sectional Model 2 Clinical Covariate Sensitivity Analysis Effects	32](#_Toc238208081)

[Supplementary Table S13. Summary of Cross-Sectional Model 3 Clinical Covariate Sensitivity Analysis Effects	33](#_Toc238208082)

[Supplementary Table S14. Summary of Longitudinal Model 1 Clinical Covariate Sensitivity Analysis Effects	34](#_Toc238208083)

[Supplementary Table S15. Summary of Longitudinal Model 2 Clinical Covariate Sensitivity Analysis Effects	35](#_Toc238208084)

[Supplementary Table S16. Summary of Longitudinal Model 3 Clinical Covariate Sensitivity Analysis Effects	36](#_Toc238208085)

[Supplementary Table S17. Summary of Continuous PGS-CRP Sensitivity Analysis Effects (Model 1)	37](#_Toc238208086)

[Supplementary Table S18. Summary of Quintile and Quadratic PGS-CRP Sensitivity Analysis Effects for Large Reward Anticipation (Model 1)	39](#_Toc238208087)

[Supplementary Figure S3. PGS-CRP × Large Reward Anticipation Interaction Estimates by PGS-CRP Quintile	41](#_Toc238208088)

[Supplementary Table S19. Parent-Reported Pubertal Development Scale Scores by Imaging Visit and Sex	42](#_Toc238208089)

[Supplementary Figure S4. Parent-Reported Pubertal Development Scale Stage by Imaging Visit and Sex	43](#_Toc238208090)

[Supplementary Table S20. Summary of Longitudinal Model 1 Adjusted for Time-Varying Pubertal Status	44](#_Toc238208091)

[Supplementary Table S21. Summary of Pubertal Status Moderation of Longitudinal Model 1	45](#_Toc238208092)

# Supplementary Table S1. Baseline Characteristics of Participants Contributing Data at Each Imaging Visit by PGS-CRP Group

|  | **Year 0** | | **Year 2** | | **Year 4** | | **Year 6** | |
| --- | --- | --- | --- | --- | --- | --- | --- | --- |
| **Characteristic** | **Low^[1]^** N = 2408 | **High^[2]^** N = 2422 | **Low** N = 1546 | **High** N = 1567 | **Low** N = 1255 | **High** N = 1186 | **Low** N = 907 | **High** N = 865 |
| **Age at visit, years** | 9.98 (0.63) | 9.96 (0.62) | 12.03 (0.65) | 12.02 (0.65) | 14.24 (0.74) | 14.19 (0.72) | 16.18 (0.66) | 16.11 (0.65) |
| **Sex, female** | 1,155 (48.0%) | 1,174 (48.5%) | 701 (45.3%) | 742 (47.4%) | 588 (46.9%) | 554 (46.7%) | 442 (48.7%) | 419 (48.4%) |
| **BMI** | 17.62 (3.00) | 18.12 (3.46) | 17.62 (3.06) | 17.99 (3.33) | 17.55 (2.98) | 18.13 (3.48) | 17.65 (3.02) | 18.07 (3.34) |
| **Household income** |  |  |  |  |  |  |  |  |
| <25k | 68 (2.8%) | 116 (4.8%) | 36 (2.3%) | 59 (3.8%) | 29 (2.3%) | 44 (3.7%) | 24 (2.6%) | 32 (3.7%) |
| 25k–50k | 186 (7.7%) | 233 (9.6%) | 131 (8.5%) | 158 (10.1%) | 99 (7.9%) | 114 (9.6%) | 73 (8.0%) | 80 (9.2%) |
| 50k–75k | 334 (13.9%) | 333 (13.7%) | 213 (13.8%) | 234 (14.9%) | 173 (13.8%) | 176 (14.8%) | 128 (14.1%) | 118 (13.6%) |
| 75k–100k | 395 (16.4%) | 413 (17.1%) | 282 (18.2%) | 275 (17.5%) | 204 (16.3%) | 207 (17.5%) | 150 (16.5%) | 151 (17.5%) |
| 100k–200k | 1,024 (42.5%) | 961 (39.7%) | 641 (41.5%) | 622 (39.7%) | 555 (44.2%) | 467 (39.4%) | 404 (44.5%) | 357 (41.3%) |
| >200k | 401 (16.7%) | 366 (15.1%) | 243 (15.7%) | 219 (14.0%) | 195 (15.5%) | 178 (15.0%) | 128 (14.1%) | 127 (14.7%) |
| **Caregiver education** |  |  |  |  |  |  |  |  |
| Up to HS (No diploma) | 8 (0.3%) | 10 (0.4%) | 6 (0.4%) | 8 (0.5%) | 5 (0.4%) | 5 (0.4%) | 4 (0.4%) | 4 (0.5%) |
| HS diploma/GED | 61 (2.5%) | 79 (3.3%) | 40 (2.6%) | 45 (2.9%) | 33 (2.6%) | 30 (2.5%) | 18 (2.0%) | 19 (2.2%) |
| Some college | 381 (15.8%) | 499 (20.6%) | 245 (15.8%) | 326 (20.8%) | 186 (14.8%) | 233 (19.6%) | 131 (14.4%) | 163 (18.8%) |
| Bachelor's degree | 785 (32.6%) | 759 (31.3%) | 506 (32.7%) | 481 (30.7%) | 415 (33.1%) | 367 (30.9%) | 313 (34.5%) | 278 (32.1%) |
| Graduate/professional | 1,173 (48.7%) | 1,075 (44.4%) | 749 (48.4%) | 707 (45.1%) | 616 (49.1%) | 551 (46.5%) | 441 (48.6%) | 401 (46.4%) |
| **Psychiatric diagnoses** |  |  |  |  |  |  |  |  |
| ADHD | 300 (12.5%) | 361 (14.9%) | 206 (13.3%) | 214 (13.7%) | 160 (12.7%) | 171 (14.4%) | 126 (13.9%) | 133 (15.4%) |
| ODD | 326 (13.5%) | 391 (16.1%) | 210 (13.6%) | 243 (15.5%) | 173 (13.8%) | 188 (15.9%) | 125 (13.8%) | 125 (14.5%) |
| MDD | 51 (2.1%) | 60 (2.5%) | 28 (1.8%) | 33 (2.1%) | 25 (2.0%) | 33 (2.8%) | 18 (2.0%) | 18 (2.1%) |
| BD^[3]^ | 49 (2.0%) | 55 (2.3%) | 37 (2.4%) | 36 (2.3%) | 25 (2.0%) | 24 (2.0%) | 19 (2.1%) | 17 (2.0%) |
| Other mood disorder^[4]^ | 102 (4.2%) | 162 (6.7%) | 68 (4.4%) | 100 (6.4%) | 50 (4.0%) | 80 (6.7%) | 37 (4.1%) | 49 (5.7%) |
| GAD | 116 (4.8%) | 127 (5.2%) | 76 (4.9%) | 77 (4.9%) | 51 (4.1%) | 60 (5.1%) | 40 (4.4%) | 48 (5.5%) |
| PTSD | 23 (1.0%) | 45 (1.9%) | 15 (1.0%) | 28 (1.8%) | 13 (1.0%) | 23 (1.9%) | 10 (1.1%) | 16 (1.8%) |
| **Any psychotropic medication** | 209 (8.7%) | 239 (9.9%) | 142 (9.2%) | 148 (9.4%) | 102 (8.1%) | 113 (9.5%) | 89 (9.8%) | 80 (9.2%) |
|  | | | | | | | | |

*Note*. Descriptive characteristics for participants contributing data at each imaging timepoint (Year 0 baseline, Year 2, Year 4, and Year 6), presented stratified by PGS-CRP group. Continuous variables (i.e., age and BMI) are presented as: mean (standard deviation). Categorical variables (all others) are presented as: frequency (percentage). The longitudinal sample was restricted to participants with complete data at Year 0; participants were not required to contribute data at more than one timepoint. Age at visit reflects the observed age at each timepoint. All other variables (BMI, household income, caregiver education, and psychiatric diagnoses) reflect baseline-measured values, facilitating assessment of differential attrition across timepoints. *Abbreviations:* PGS-CRP = polygenic score for C-reactive protein; BMI = body mass index; ADHD = attention-deficit/hyperactivity disorder; ODD = oppositional defiant disorder; MDD = major depressive disorder; BD = bipolar disorder; GAD = generalized anxiety disorder; PTSD = post-traumatic stress disorder.

# Supplementary Figure S1. Distribution of PGS-CRP Scores in the Baseline Analytical Sample

*Note.* Histogram of polygenic score for C-reactive protein (PGS-CRP) values in the European-ancestry analytical sample at baseline. Bar colors distinguish participants in the low and high PGS-CRP groups based on a median split. The dashed vertical line indicates the sample median. *Abbreviations:* PGS-CRP = polygenic score for C-reactive protein

# Supplementary Table S2. Caregiver-Reported Psychiatric Diagnoses by Imaging Visit and PGS-CRP Group

|  | **Year 0** | | **Year 2** | | **Year 4** | | **Year 6** | |
| --- | --- | --- | --- | --- | --- | --- | --- | --- |
| **Diagnosis** | **Low^[5]^** N = 2408 | **High^[6]^** N = 2422 | **Low** N = 1545 | **High** N = 1567 | **Low** N = 1102 | **High** N = 1045 | **Low** N = 894 | **High** N = 853 |
| **Any externalizing disorder** | 517 (21.5%) | 601 (24.8%)** | 319 (20.6%) | 357 (22.8%) | 149 (13.5%) | 149 (14.3%) | 109 (12.2%) | 119 (14.0%) |
| ADHD | 300 (12.5%) | 361 (14.9%)* | 190 (12.3%) | 215 (13.7%) | 100 (9.1%) | 90 (8.6%) | 84 (9.4%) | 88 (10.3%) |
| ODD | 326 (13.5%) | 391 (16.1%)* | 208 (13.5%) | 231 (14.7%) | 71 (6.4%) | 68 (6.5%) | -- | -- |
| Conduct disorder | 52 (2.2%) | 59 (2.4%) | 31 (2.0%) | 38 (2.4%) | 24 (2.2%) | 26 (2.5%) | 29 (3.2%) | 34 (4.0%) |
| Other externalizing disorder^[7]^ | 11 (0.5%) | 13 (0.5%) | 2 (0.1%) | 7 (0.4%) | 4 (0.4%) | 4 (0.4%) | 7 (0.8%) | 9 (1.1%) |
| **Any anxiety disorder** | 989 (41.1%) | 990 (40.9%) | 547 (35.4%) | 560 (35.7%) | 387 (35.1%) | 372 (35.6%) | 158 (17.7%) | 150 (17.6%) |
| GAD | 116 (4.8%) | 127 (5.2%) | 99 (6.4%) | 81 (5.2%) | 58 (5.3%) | 56 (5.4%) | 54 (6.0%) | 50 (5.9%) |
| OCD | 194 (8.1%) | 185 (7.6%) | 110 (7.1%) | 106 (6.8%) | 31 (2.8%) | 48 (4.6%)* | 38 (4.3%) | 35 (4.1%) |
| Social anxiety disorder | 125 (5.2%) | 127 (5.2%) | 80 (5.2%) | 81 (5.2%) | 60 (5.4%) | 68 (6.5%) | 60 (6.7%) | 72 (8.4%) |
| Separation anxiety disorder | 229 (9.5%) | 243 (10.0%) | 92 (6.0%) | 96 (6.1%) | -- | -- | -- | -- |
| Agoraphobia | 4 (0.2%) | 8 (0.3%) | 24 (1.6%) | 13 (0.8%) | 24 (2.2%) | 25 (2.4%) | 19 (2.1%) | 14 (1.6%) |
| Specific phobia | 621 (25.8%) | 632 (26.1%) | 348 (22.5%) | 348 (22.2%) | 316 (28.7%) | 264 (25.3%) | -- | -- |
| Panic disorder | 9 (0.4%) | 3 (0.1%) | 6 (0.4%) | 3 (0.2%) | 20 (1.8%) | 19 (1.8%) | 28 (3.1%) | 25 (2.9%) |
| Other anxiety disorder^[8]^ | 270 (11.2%) | 271 (11.2%) | 133 (8.6%) | 150 (9.6%) | 59 (5.4%) | 45 (4.3%) | 48 (5.4%) | 34 (4.0%) |
| **Any autism spectrum disorder** | 605 (25.1%) | 632 (26.1%) | 322 (20.8%) | 303 (19.3%) | 14 (1.3%) | 12 (1.1%) | -- | -- |
| ASD | -- | -- | -- | -- | 0 (0.0%) | 0 (0.0%) | -- | -- |
| Other ASD-related disorder^[9]^ | 605 (25.1%) | 632 (26.1%) | 322 (20.8%) | 303 (19.3%) | 14 (1.3%) | 12 (1.1%) | -- | -- |
| **Any mood disorder** | 203 (8.4%) | 268 (11.1%)** | 125 (8.1%) | 171 (10.9%)** | 80 (7.3%) | 87 (8.3%) | 79 (8.8%) | 73 (8.6%) |
| MDD | 51 (2.1%) | 60 (2.5%) | 44 (2.8%) | 61 (3.9%) | 78 (7.1%) | 85 (8.1%) | 76 (8.5%) | 70 (8.2%) |
| PDD | 3 (0.1%) | 3 (0.1%) | 2 (0.1%) | 2 (0.1%) | 6 (0.5%) | 9 (0.9%) | 6 (0.7%) | 7 (0.8%) |
| DMDD | 3 (0.1%) | 1 (0.0%) | 0 (0.0%) | 1 (0.1%) | -- | -- | -- | -- |
| BD | 49 (2.0%) | 55 (2.3%) | 21 (1.4%) | 23 (1.5%) | 2 (0.2%) | 1 (0.1%) | 2 (0.2%) | 1 (0.1%) |
| Other mood disorder^[10]^ | 102 (4.2%) | 162 (6.7%)*** | 66 (4.3%) | 92 (5.9%) | 1 (0.1%) | 1 (0.1%) | 1 (0.1%) | 2 (0.2%) |
| **Any psychotic disorder** | 41 (1.7%) | 55 (2.3%) | 28 (1.8%) | 40 (2.6%)* | 4 (0.4%) | 0 (0.0%) | 1 (0.1%) | 0 (0.0%) |
| Schizophrenia | -- | -- | -- | -- | 2 (0.2%) | 0 (0.0%) | 1 (0.1%) | 0 (0.0%) |
| Schizoaffective disorder | -- | -- | -- | -- | 0 (0.0%) | 0 (0.0%) | 0 (0.0%) | 0 (0.0%) |
| Schizophreniform disorder | -- | -- | -- | -- | 0 (0.0%) | 0 (0.0%) | 0 (0.0%) | 0 (0.0%) |
| Other psychotic disorder^[11]^ | 41 (1.7%) | 55 (2.3%) | 28 (1.8%) | 40 (2.6%) | 2 (0.2%) | 0 (0.0%) | 0 (0.0%) | 0 (0.0%) |
| **Any stress-related disorder** | 70 (2.9%) | 111 (4.6%)** | 57 (3.7%) | 61 (3.9%) | 37 (3.4%) | 39 (3.7%) | 56 (6.3%) | 55 (6.4%) |
| PTSD | 23 (1.0%) | 45 (1.9%)** | 27 (1.7%) | 30 (1.9%) | 28 (2.5%) | 29 (2.8%) | 35 (3.9%) | 41 (4.8%) |
| Other stress-related disorder^[12]^ | 48 (2.0%) | 70 (2.9%)* | 33 (2.1%) | 33 (2.1%) | 9 (0.8%) | 12 (1.1%) | 23 (2.6%) | 16 (1.9%) |
| **Any tic disorder** | -- | -- | -- | -- | -- | -- | 51 (5.7%) | 54 (6.3%) |
| Tourette's disorder | -- | -- | -- | -- | -- | -- | 4 (0.4%) | 6 (0.7%) |
| Persistent tic disorder | -- | -- | -- | -- | -- | -- | 37 (4.1%) | 35 (4.1%) |
| Provisional tic disorder | -- | -- | -- | -- | -- | -- | 13 (1.5%) | 16 (1.9%) |
| Other tic disorder^[13]^ | -- | -- | -- | -- | -- | -- | -- | -- |

*Note.* Frequencies and percentages of all caregiver-reported psychiatric diagnoses from the computerized version of the Kiddie Schedule for Affective Disorders and Schizophrenia (KSADS-COMP) at each imaging visit, presented by PGS-CRP group. Diagnosis categories comprise both past and present diagnoses. Individual diagnoses within each diagnostic domain and domain-level summary variables are included. Dashes indicate that the corresponding KSADS-COMP module was not administered at that visit. Asterisks indicate statistical significance between PGS-CRP groups using Fisher’s exact test: *** = p < 0.001; ** = p < 0.01; * = p < 0.05. *Abbreviations:* KSADS-COMP = Kiddie Schedule for Affective Disorders and Schizophrenia, computerized version; PGS-CRP = polygenic score for C-reactive protein; ADHD = attention-deficit/hyperactivity disorder; ODD = oppositional defiant disorder; GAD = generalized anxiety disorder; OCD = obsessive-compulsive disorder; ASD = autism spectrum disorder; MDD = major depressive disorder; PDD = persistent depressive disorder; DMDD = disruptive mood dysregulation disorder; BD = bipolar disorder; PTSD = post-traumatic stress disorder.

# Supplementary Table S3. Caregiver-Reported Psychotropic Medication Use by Imaging Visit and PGS-CRP Group

|  | **Year 0** | | **Year 2** | | **Year 4** | | **Year 6** | |
| --- | --- | --- | --- | --- | --- | --- | --- | --- |
| **Medication** | **Low^[14]^** N = 2407 | **High^[15]^** N = 2419 | **Low** N = 1536 | **High** N = 1559 | **Low** N = 1251 | **High** N = 1181 | **Low** N = 898 | **High** N = 862 |
| **Any psychotropic medication** | 209 (8.7%) | 239 (9.9%) | 179 (11.7%) | 201 (12.9%) | 212 (16.9%) | 217 (18.4%) | 209 (23.3%) | 206 (23.9%) |
| **ADHD medication** |  |  |  |  |  |  |  |  |
| Amphetamine | 61 (2.5%) | 61 (2.5%) | 60 (3.9%) | 50 (3.2%) | 52 (4.2%) | 58 (4.9%) | 48 (5.3%) | 45 (5.2%) |
| Methylphenidate | 99 (4.1%) | 125 (5.2%) | 70 (4.6%) | 91 (5.8%) | 59 (4.7%) | 80 (6.8%)* | 57 (6.3%) | 73 (8.5%) |
| Alpha-2 adrenergic agonist | 47 (2.0%) | 51 (2.1%) | 39 (2.5%) | 44 (2.8%) | 33 (2.6%) | 27 (2.3%) | 24 (2.7%) | 23 (2.7%) |
| Norepinephrine reuptake inhibitor | 9 (0.4%) | 11 (0.5%) | 7 (0.5%) | 11 (0.7%) | 6 (0.5%) | 6 (0.5%) | 6 (0.7%) | 8 (0.9%) |
| **Antidepressants** |  |  |  |  |  |  |  |  |
| Non-SSRI antidepressant | 10 (0.4%) | 9 (0.4%) | 11 (0.7%) | 12 (0.8%) | 25 (2.0%) | 24 (2.0%) | 41 (4.6%) | 43 (5.0%) |
| SSRI | 46 (1.9%) | 50 (2.1%) | 47 (3.1%) | 54 (3.5%) | 98 (7.8%) | 97 (8.2%) | 116 (12.9%) | 100 (11.6%) |
| **Antipsychotics/Mood stabilizers** |  |  |  |  |  |  |  |  |
| Antipsychotic | 13 (0.5%) | 10 (0.4%) | 11 (0.7%) | 10 (0.6%) | 15 (1.2%) | 19 (1.6%) | 17 (1.9%) | 14 (1.6%) |
| Mood stabilizer | 4 (0.2%) | 4 (0.2%) | 11 (0.7%) | 10 (0.6%) | 16 (1.3%) | 11 (0.9%) | 14 (1.6%) | 14 (1.6%) |
| **Sedative-hypnotics/Anxiolytics** |  |  |  |  |  |  |  |  |
| Hypnotic/sleep aid | 3 (0.1%) | 11 (0.5%) | 2 (0.1%) | 6 (0.4%) | 2 (0.2%) | 0 (0.0%) | 0 (0.0%) | 1 (0.1%) |
| Anxiolytic | 1 (0.0%) | 1 (0.0%) | 1 (0.1%) | 5 (0.3%) | 10 (0.8%) | 3 (0.3%) | 8 (0.9%) | 13 (1.5%) |
| Alpha-1 blocker (PTSD) | 0 (0.0%) | 1 (0.0%) | 0 (0.0%) | 1 (0.1%) | 2 (0.2%) | 0 (0.0%) | 2 (0.2%) | 2 (0.2%) |

*Note.* Frequencies and percentages of caregiver-reported psychotropic medication use in the two weeks prior to each imaging visit, by medication class and PGS-CRP group. Medication classifications based on previous ABCD work (Lopez et al., 2026). Medication use reflects prescription medications recorded in the medication inventory. Asterisks indicate statistical significance between PGS-CRP groups using Fisher’s exact test: *** = p < 0.001; ** = p < 0.01; * = p < 0.05. *Abbreviations:* PGS-CRP = polygenic score for C-reactive protein; ADHD = attention-deficit/hyperactivity disorder; SSRI = selective-serotonin reuptake inhibitor; PTSD = post-traumatic stress disorder.

# Supplementary Table S4. Complete Fixed-Effect Output for Cross-Sectional Model 1

| **ROI** | **Term** | **β** | **95% CI** | ***p*** | ***p*_FDR_** |
| --- | --- | --- | --- | --- | --- |
| Caudate | Age at baseline | 0.003 | [-0.006, 0.012] | 0.556 | 0.833 |
| Caudate | BMI | -0.006 | [-0.011, 0.000] | 0.053 | 0.090 |
| Caudate | Caregiver edu: Bachelor's | 0.071 | [-0.023, 0.164] | 0.138 | 0.207 |
| Caudate | Caregiver edu: Graduate/professional | 0.074 | [-0.019, 0.168] | 0.119 | 0.179 |
| Caudate | Caregiver edu: HS/GED | 0.049 | [-0.048, 0.146] | 0.324 | 0.485 |
| Caudate | Caregiver edu: Some college | 0.060 | [-0.033, 0.153] | 0.209 | 0.314 |
| Caudate | Condition (large loss) | 0.031 | [0.021, 0.041] | <.001 | <.001 |
| Caudate | Condition (large win) | 0.082 | [0.072, 0.092] | <.001 | <.001 |
| Caudate | Condition (small loss) | -0.015 | [-0.025, -0.006] | 0.002 | 0.002 |
| Caudate | Income: 100k–200k | -0.020 | [-0.053, 0.012] | 0.225 | 0.337 |
| Caudate | Income: 25k–50k | -0.024 | [-0.059, 0.011] | 0.175 | 0.259 |
| Caudate | Income: 50k–75k | -0.016 | [-0.049, 0.018] | 0.360 | 0.540 |
| Caudate | Income: 75k–100k | -0.009 | [-0.042, 0.025] | 0.612 | 0.918 |
| Caudate | Income: >200k | -0.024 | [-0.059, 0.011] | 0.183 | 0.275 |
| Caudate | Intercept | -0.047 | [-0.177, 0.083] | 0.479 | 0.479 |
| Caudate | PC1 | 0.003 | [-0.005, 0.011] | 0.450 | 0.675 |
| Caudate | PC2 | -0.003 | [-0.010, 0.003] | 0.338 | 0.338 |
| Caudate | PC3 | -0.005 | [-0.011, 0.001] | 0.115 | 0.115 |
| Caudate | PC4 | -0.002 | [-0.011, 0.006] | 0.591 | 0.591 |
| Caudate | PC5 | -0.001 | [-0.010, 0.008] | 0.818 | 0.861 |
| Caudate | PC6 | 0.005 | [-0.003, 0.012] | 0.222 | 0.222 |
| Caudate | PC7 | -0.001 | [-0.008, 0.006] | 0.803 | 0.960 |
| Caudate | PC8 | 0.005 | [-0.004, 0.014] | 0.242 | 0.520 |
| Caudate | PC9 | -0.007 | [-0.014, -0.000] | 0.040 | 0.071 |
| Caudate | PC10 | -0.002 | [-0.011, 0.006] | 0.607 | 0.796 |
| Caudate | PGS-CRP | 0.001 | [-0.013, 0.015] | 0.853 | 0.862 |
| Caudate | PGS-CRP x Large Loss | -0.009 | [-0.022, 0.005] | 0.223 | 0.381 |
| Caudate | PGS-CRP x Large Win | -0.017 | [-0.031, -0.004] | 0.013 | 0.022 |
| Caudate | PGS-CRP x Small Loss | 0.002 | [-0.012, 0.016] | 0.779 | 0.779 |
| Caudate | Scanner: Philips | -0.006 | [-0.039, 0.027] | 0.722 | 0.939 |
| Caudate | Scanner: Siemens | -0.006 | [-0.028, 0.015] | 0.578 | 0.578 |
| Caudate | Sex (Female) | 0.004 | [-0.007, 0.016] | 0.449 | 0.949 |
| Nucleus Accumbens | Age at baseline | -0.001 | [-0.010, 0.009] | 0.900 | 0.900 |
| Nucleus Accumbens | BMI | -0.005 | [-0.011, 0.001] | 0.133 | 0.133 |
| Nucleus Accumbens | Caregiver edu: Bachelor's | 0.112 | [0.014, 0.210] | 0.025 | 0.075 |
| Nucleus Accumbens | Caregiver edu: Graduate/professional | 0.112 | [0.014, 0.209] | 0.025 | 0.076 |
| Nucleus Accumbens | Caregiver edu: HS/GED | 0.075 | [-0.027, 0.177] | 0.149 | 0.446 |
| Nucleus Accumbens | Caregiver edu: Some college | 0.100 | [0.002, 0.197] | 0.045 | 0.135 |
| Nucleus Accumbens | Condition (large loss) | -0.017 | [-0.027, -0.006] | 0.002 | 0.002 |
| Nucleus Accumbens | Condition (large win) | 0.080 | [0.070, 0.091] | <.001 | <.001 |
| Nucleus Accumbens | Condition (small loss) | -0.031 | [-0.041, -0.021] | <.001 | <.001 |
| Nucleus Accumbens | Income: 100k–200k | -0.028 | [-0.062, 0.006] | 0.109 | 0.327 |
| Nucleus Accumbens | Income: 25k–50k | -0.023 | [-0.059, 0.014] | 0.228 | 0.259 |
| Nucleus Accumbens | Income: 50k–75k | -0.029 | [-0.064, 0.006] | 0.108 | 0.324 |
| Nucleus Accumbens | Income: 75k–100k | -0.017 | [-0.052, 0.018] | 0.351 | 0.918 |
| Nucleus Accumbens | Income: >200k | -0.028 | [-0.065, 0.008] | 0.132 | 0.275 |
| Nucleus Accumbens | Intercept | -0.054 | [-0.189, 0.080] | 0.428 | 0.479 |
| Nucleus Accumbens | PC1 | -0.004 | [-0.011, 0.004] | 0.379 | 0.675 |
| Nucleus Accumbens | PC2 | 0.005 | [-0.002, 0.012] | 0.190 | 0.338 |
| Nucleus Accumbens | PC3 | -0.006 | [-0.012, 0.001] | 0.089 | 0.115 |
| Nucleus Accumbens | PC4 | -0.004 | [-0.013, 0.005] | 0.354 | 0.532 |
| Nucleus Accumbens | PC5 | -0.001 | [-0.010, 0.008] | 0.861 | 0.861 |
| Nucleus Accumbens | PC6 | 0.008 | [0.000, 0.016] | 0.047 | 0.070 |
| Nucleus Accumbens | PC7 | -0.001 | [-0.008, 0.007] | 0.853 | 0.960 |
| Nucleus Accumbens | PC8 | 0.004 | [-0.005, 0.014] | 0.346 | 0.520 |
| Nucleus Accumbens | PC9 | -0.003 | [-0.010, 0.004] | 0.335 | 0.335 |
| Nucleus Accumbens | PC10 | -0.001 | [-0.010, 0.008] | 0.796 | 0.796 |
| Nucleus Accumbens | PGS-CRP | 0.007 | [-0.008, 0.022] | 0.373 | 0.862 |
| Nucleus Accumbens | PGS-CRP x Large Loss | -0.007 | [-0.021, 0.008] | 0.381 | 0.381 |
| Nucleus Accumbens | PGS-CRP x Large Win | -0.017 | [-0.032, -0.002] | 0.022 | 0.022 |
| Nucleus Accumbens | PGS-CRP x Small Loss | -0.004 | [-0.018, 0.011] | 0.610 | 0.779 |
| Nucleus Accumbens | Scanner: Philips | 0.003 | [-0.019, 0.025] | 0.801 | 0.939 |
| Nucleus Accumbens | Scanner: Siemens | 0.012 | [-0.004, 0.027] | 0.150 | 0.450 |
| Nucleus Accumbens | Sex (Female) | -0.000 | [-0.012, 0.011] | 0.949 | 0.949 |
| Putamen | Age at baseline | 0.005 | [-0.002, 0.013] | 0.161 | 0.484 |
| Putamen | BMI | -0.005 | [-0.009, 0.000] | 0.060 | 0.090 |
| Putamen | Caregiver edu: Bachelor's | 0.023 | [-0.055, 0.101] | 0.557 | 0.557 |
| Putamen | Caregiver edu: Graduate/professional | 0.027 | [-0.051, 0.105] | 0.499 | 0.499 |
| Putamen | Caregiver edu: HS/GED | 0.014 | [-0.067, 0.096] | 0.728 | 0.728 |
| Putamen | Caregiver edu: Some college | 0.016 | [-0.062, 0.093] | 0.691 | 0.691 |
| Putamen | Condition (large loss) | 0.009 | [0.001, 0.017] | 0.026 | 0.026 |
| Putamen | Condition (large win) | 0.057 | [0.049, 0.065] | <.001 | <.001 |
| Putamen | Condition (small loss) | -0.016 | [-0.024, -0.008] | <.001 | <.001 |
| Putamen | Income: 100k–200k | -0.005 | [-0.032, 0.022] | 0.711 | 0.711 |
| Putamen | Income: 25k–50k | -0.017 | [-0.046, 0.012] | 0.259 | 0.259 |
| Putamen | Income: 50k–75k | -0.002 | [-0.030, 0.026] | 0.913 | 0.913 |
| Putamen | Income: 75k–100k | 0.001 | [-0.027, 0.028] | 0.972 | 0.972 |
| Putamen | Income: >200k | -0.008 | [-0.037, 0.021] | 0.589 | 0.589 |
| Putamen | Intercept | -0.053 | [-0.162, 0.055] | 0.337 | 0.479 |
| Putamen | PC1 | 0.001 | [-0.005, 0.007] | 0.768 | 0.768 |
| Putamen | PC2 | -0.003 | [-0.009, 0.003] | 0.304 | 0.338 |
| Putamen | PC3 | -0.005 | [-0.010, 0.001] | 0.082 | 0.115 |
| Putamen | PC4 | -0.004 | [-0.011, 0.003] | 0.273 | 0.532 |
| Putamen | PC5 | 0.002 | [-0.006, 0.009] | 0.693 | 0.861 |
| Putamen | PC6 | 0.007 | [0.000, 0.013] | 0.040 | 0.070 |
| Putamen | PC7 | -0.000 | [-0.006, 0.006] | 0.960 | 0.960 |
| Putamen | PC8 | 0.002 | [-0.005, 0.009] | 0.555 | 0.555 |
| Putamen | PC9 | -0.006 | [-0.011, -0.000] | 0.048 | 0.071 |
| Putamen | PC10 | -0.004 | [-0.011, 0.003] | 0.220 | 0.661 |
| Putamen | PGS-CRP | 0.001 | [-0.011, 0.013] | 0.862 | 0.862 |
| Putamen | PGS-CRP x Large Loss | -0.006 | [-0.017, 0.006] | 0.334 | 0.381 |
| Putamen | PGS-CRP x Large Win | -0.014 | [-0.025, -0.002] | 0.021 | 0.022 |
| Putamen | PGS-CRP x Small Loss | 0.002 | [-0.010, 0.013] | 0.743 | 0.779 |
| Putamen | Scanner: Philips | -0.001 | [-0.026, 0.024] | 0.939 | 0.939 |
| Putamen | Scanner: Siemens | -0.009 | [-0.025, 0.008] | 0.318 | 0.477 |
| Putamen | Sex (Female) | -0.001 | [-0.010, 0.009] | 0.861 | 0.949 |

*Note.* All fixed-effect estimates from the cross-sectional Model 1, including all covariate terms. Body mass index (BMI) and principal genetic components (PC1-10) are scaled in the model. *Abbreviations:* ROI = region of interest; β = beta coefficient representing the difference in percent signal change between High and Low PGS-CRP groups; 95% CI = 95% confidence interval; *p*_FDR_ = Benjamini-Hochberg false discovery rate-corrected p-value; BMI = body mass index; PGS-CRP = polygenic score for C-reactive protein.

# Supplementary Table S5. Summary of Model 1 Longitudinal Sensitivity Analysis Effects

| **ROI** | **Term** | **β** | **95% CI** | ***p*** | ***p*_FDR_** |
| --- | --- | --- | --- | --- | --- |
| Caudate | Condition (large loss) | 0.045 | [0.038, 0.053] | <.001 | <.001 |
| Caudate | Condition (large win) | 0.096 | [0.089, 0.103] | <.001 | <.001 |
| Caudate | Condition (small loss) | -0.005 | [-0.012, 0.002] | 0.186 | 0.186 |
| Caudate | Intercept | -0.059 | [-0.138, 0.020] | 0.145 | 0.145 |
| Caudate | PGS-CRP | 0.003 | [-0.007, 0.013] | 0.514 | 0.771 |
| Caudate | PGS-CRP x Large Loss | -0.003 | [-0.014, 0.007] | 0.520 | 0.780 |
| Caudate | PGS-CRP x Large Win | -0.013 | [-0.024, -0.003] | 0.011 | 0.032 |
| Caudate | PGS-CRP x Small Loss | -0.001 | [-0.011, 0.010] | 0.892 | 0.892 |
| Nucleus Accumbens | Condition (large loss) | -0.005 | [-0.013, 0.003] | 0.230 | 0.230 |
| Nucleus Accumbens | Condition (large win) | 0.084 | [0.076, 0.092] | <.001 | <.001 |
| Nucleus Accumbens | Condition (small loss) | -0.015 | [-0.024, -0.007] | <.001 | <.001 |
| Nucleus Accumbens | Intercept | -0.133 | [-0.224, -0.041] | 0.005 | 0.014 |
| Nucleus Accumbens | PGS-CRP | 0.007 | [-0.005, 0.018] | 0.259 | 0.771 |
| Nucleus Accumbens | PGS-CRP x Large Loss | -0.010 | [-0.022, 0.002] | 0.099 | 0.297 |
| Nucleus Accumbens | PGS-CRP x Large Win | -0.011 | [-0.022, 0.001] | 0.070 | 0.078 |
| Nucleus Accumbens | PGS-CRP x Small Loss | -0.007 | [-0.019, 0.005] | 0.244 | 0.593 |
| Putamen | Condition (large loss) | 0.017 | [0.011, 0.023] | <.001 | <.001 |
| Putamen | Condition (large win) | 0.065 | [0.059, 0.071] | <.001 | <.001 |
| Putamen | Condition (small loss) | -0.012 | [-0.018, -0.006] | <.001 | <.001 |
| Putamen | Intercept | -0.063 | [-0.129, 0.002] | 0.058 | 0.087 |
| Putamen | PGS-CRP | -0.000 | [-0.009, 0.008] | 0.927 | 0.927 |
| Putamen | PGS-CRP x Large Loss | -0.001 | [-0.010, 0.007] | 0.794 | 0.794 |
| Putamen | PGS-CRP x Large Win | -0.008 | [-0.016, 0.001] | 0.078 | 0.078 |
| Putamen | PGS-CRP x Small Loss | 0.004 | [-0.005, 0.012] | 0.396 | 0.593 |

*Note.* Fixed-effect estimates from longitudinal Model 1 across four imaging timepoints. Age was decomposed into baseline age and within-person age change from baseline. Abbreviations: ROI = region of interest; β = beta coefficient; CI = confidence interval; *p*_FDR_ = Benjamini–Hochberg false discovery rate-corrected p-value; PGS-CRP = polygenic score for C-reactive protein.

# Supplementary Figure S2. Longitudinal Sensitivity Analysis (Models 1 and 2): PGS-CRP Group Differences in Striatal Activation During Reward and Loss Anticipation in the MID Task

*Note.* Estimated marginal means (EMMs) for striatal activation during anticipation conditions of the Monetary Incentive Delay (MID) task, relative to the neutral anticipation condition, for low and high PGS-CRP groups in the nucleus accumbens, caudate, and putamen. (A) Longitudinal model 1 results. (B) Longitudinal model 2 results. Error bars represent 95% confidence intervals. Asterisks denote False Discovery Rate (FDR)-corrected significant effects (*p*_FDR_ < 0.05). Abbreviations: EMMs = estimated marginal means; MID = Monetary Incentive Delay; *p*_FDR_ = Benjamini–Hochberg false discovery rate-corrected p-value; PGS-CRP = polygenic score for C-reactive protein.

# Supplementary Table S6. Complete Fixed-Effect Output for Cross-Sectional Model 2

| **ROI** | **Term** | **β** | **95% CI** | ***p*** | ***p*_FDR_** |
| --- | --- | --- | --- | --- | --- |
| Caudate | Age at baseline | 0.003 | [-0.006, 0.012] | 0.542 | 0.813 |
| Caudate | BMI | -0.006 | [-0.011, 0.000] | 0.054 | 0.092 |
| Caudate | Caregiver edu: Bachelor's | 0.071 | [-0.022, 0.164] | 0.137 | 0.206 |
| Caudate | Caregiver edu: Graduate/professional | 0.074 | [-0.019, 0.168] | 0.118 | 0.177 |
| Caudate | Caregiver edu: HS/GED | 0.049 | [-0.049, 0.146] | 0.328 | 0.492 |
| Caudate | Caregiver edu: Some college | 0.059 | [-0.034, 0.152] | 0.211 | 0.316 |
| Caudate | Condition (loss) | -0.033 | [-0.041, -0.026] | <.001 | <.001 |
| Caudate | Income: 100k–200k | -0.021 | [-0.053, 0.012] | 0.218 | 0.327 |
| Caudate | Income: 25k–50k | -0.024 | [-0.059, 0.011] | 0.175 | 0.266 |
| Caudate | Income: 50k–75k | -0.016 | [-0.049, 0.018] | 0.359 | 0.538 |
| Caudate | Income: 75k–100k | -0.009 | [-0.042, 0.025] | 0.609 | 0.913 |
| Caudate | Income: >200k | -0.024 | [-0.059, 0.011] | 0.178 | 0.266 |
| Caudate | Intercept | -0.007 | [-0.137, 0.123] | 0.917 | 0.917 |
| Caudate | PC1 | 0.003 | [-0.005, 0.011] | 0.446 | 0.669 |
| Caudate | PC2 | -0.003 | [-0.010, 0.003] | 0.342 | 0.342 |
| Caudate | PC3 | -0.005 | [-0.011, 0.001] | 0.108 | 0.108 |
| Caudate | PC4 | -0.002 | [-0.011, 0.006] | 0.579 | 0.579 |
| Caudate | PC5 | -0.001 | [-0.010, 0.008] | 0.794 | 0.884 |
| Caudate | PC6 | 0.005 | [-0.003, 0.013] | 0.208 | 0.208 |
| Caudate | PC7 | -0.001 | [-0.008, 0.007] | 0.832 | 0.974 |
| Caudate | PC8 | 0.005 | [-0.003, 0.014] | 0.220 | 0.546 |
| Caudate | PC9 | -0.007 | [-0.014, -0.000] | 0.040 | 0.067 |
| Caudate | PC10 | -0.002 | [-0.011, 0.006] | 0.579 | 0.841 |
| Caudate | PGS-CRP | -0.007 | [-0.020, 0.005] | 0.241 | 0.440 |
| Caudate | PGS-CRP x Loss | 0.005 | [-0.005, 0.016] | 0.289 | 0.434 |
| Caudate | Scanner: Philips | -0.006 | [-0.039, 0.027] | 0.717 | 0.921 |
| Caudate | Scanner: Siemens | -0.006 | [-0.028, 0.015] | 0.578 | 0.578 |
| Caudate | Sex (Female) | 0.005 | [-0.007, 0.016] | 0.430 | 0.912 |
| Nucleus Accumbens | Age at baseline | -0.001 | [-0.010, 0.009] | 0.898 | 0.898 |
| Nucleus Accumbens | BMI | -0.005 | [-0.011, 0.001] | 0.132 | 0.132 |
| Nucleus Accumbens | Caregiver edu: Bachelor's | 0.111 | [0.014, 0.209] | 0.026 | 0.077 |
| Nucleus Accumbens | Caregiver edu: Graduate/professional | 0.111 | [0.014, 0.209] | 0.026 | 0.078 |
| Nucleus Accumbens | Caregiver edu: HS/GED | 0.077 | [-0.025, 0.179] | 0.140 | 0.419 |
| Nucleus Accumbens | Caregiver edu: Some college | 0.100 | [0.002, 0.197] | 0.046 | 0.137 |
| Nucleus Accumbens | Condition (loss) | -0.064 | [-0.071, -0.056] | <.001 | <.001 |
| Nucleus Accumbens | Income: 100k–200k | -0.027 | [-0.062, 0.007] | 0.116 | 0.327 |
| Nucleus Accumbens | Income: 25k–50k | -0.022 | [-0.059, 0.014] | 0.236 | 0.266 |
| Nucleus Accumbens | Income: 50k–75k | -0.028 | [-0.064, 0.007] | 0.114 | 0.342 |
| Nucleus Accumbens | Income: 75k–100k | -0.016 | [-0.051, 0.019] | 0.363 | 0.913 |
| Nucleus Accumbens | Income: >200k | -0.028 | [-0.064, 0.009] | 0.139 | 0.266 |
| Nucleus Accumbens | Intercept | -0.014 | [-0.149, 0.121] | 0.838 | 0.917 |
| Nucleus Accumbens | PC1 | -0.004 | [-0.012, 0.004] | 0.370 | 0.669 |
| Nucleus Accumbens | PC2 | 0.005 | [-0.003, 0.012] | 0.209 | 0.342 |
| Nucleus Accumbens | PC3 | -0.006 | [-0.012, 0.001] | 0.098 | 0.108 |
| Nucleus Accumbens | PC4 | -0.004 | [-0.012, 0.005] | 0.387 | 0.579 |
| Nucleus Accumbens | PC5 | -0.001 | [-0.010, 0.009] | 0.884 | 0.884 |
| Nucleus Accumbens | PC6 | 0.008 | [-0.000, 0.016] | 0.054 | 0.080 |
| Nucleus Accumbens | PC7 | -0.001 | [-0.008, 0.007] | 0.839 | 0.974 |
| Nucleus Accumbens | PC8 | 0.004 | [-0.005, 0.013] | 0.370 | 0.546 |
| Nucleus Accumbens | PC9 | -0.003 | [-0.010, 0.004] | 0.334 | 0.334 |
| Nucleus Accumbens | PC10 | -0.001 | [-0.010, 0.008] | 0.841 | 0.841 |
| Nucleus Accumbens | PGS-CRP | -0.001 | [-0.014, 0.012] | 0.843 | 0.843 |
| Nucleus Accumbens | PGS-CRP x Loss | 0.003 | [-0.008, 0.014] | 0.592 | 0.592 |
| Nucleus Accumbens | Scanner: Philips | 0.003 | [-0.020, 0.025] | 0.821 | 0.921 |
| Nucleus Accumbens | Scanner: Siemens | 0.011 | [-0.004, 0.026] | 0.164 | 0.451 |
| Nucleus Accumbens | Sex (Female) | -0.001 | [-0.012, 0.011] | 0.912 | 0.912 |
| Putamen | Age at baseline | 0.005 | [-0.002, 0.013] | 0.158 | 0.474 |
| Putamen | BMI | -0.005 | [-0.009, 0.000] | 0.061 | 0.092 |
| Putamen | Caregiver edu: Bachelor's | 0.023 | [-0.055, 0.101] | 0.559 | 0.559 |
| Putamen | Caregiver edu: Graduate/professional | 0.027 | [-0.051, 0.105] | 0.499 | 0.499 |
| Putamen | Caregiver edu: HS/GED | 0.015 | [-0.066, 0.096] | 0.715 | 0.715 |
| Putamen | Caregiver edu: Some college | 0.015 | [-0.062, 0.093] | 0.698 | 0.698 |
| Putamen | Condition (loss) | -0.032 | [-0.038, -0.026] | <.001 | <.001 |
| Putamen | Income: 100k–200k | -0.005 | [-0.032, 0.022] | 0.720 | 0.720 |
| Putamen | Income: 25k–50k | -0.016 | [-0.046, 0.013] | 0.266 | 0.266 |
| Putamen | Income: 50k–75k | -0.001 | [-0.029, 0.027] | 0.931 | 0.931 |
| Putamen | Income: 75k–100k | 0.001 | [-0.027, 0.029] | 0.955 | 0.955 |
| Putamen | Income: >200k | -0.008 | [-0.037, 0.021] | 0.597 | 0.597 |
| Putamen | Intercept | -0.025 | [-0.133, 0.083] | 0.651 | 0.917 |
| Putamen | PC1 | 0.001 | [-0.005, 0.007] | 0.775 | 0.775 |
| Putamen | PC2 | -0.003 | [-0.009, 0.003] | 0.290 | 0.342 |
| Putamen | PC3 | -0.005 | [-0.010, 0.001] | 0.080 | 0.108 |
| Putamen | PC4 | -0.004 | [-0.011, 0.003] | 0.273 | 0.579 |
| Putamen | PC5 | 0.002 | [-0.006, 0.009] | 0.687 | 0.884 |
| Putamen | PC6 | 0.007 | [0.000, 0.013] | 0.039 | 0.080 |
| Putamen | PC7 | -0.000 | [-0.006, 0.006] | 0.974 | 0.974 |
| Putamen | PC8 | 0.002 | [-0.005, 0.009] | 0.546 | 0.546 |
| Putamen | PC9 | -0.006 | [-0.011, -0.000] | 0.044 | 0.067 |
| Putamen | PC10 | -0.005 | [-0.012, 0.003] | 0.209 | 0.628 |
| Putamen | PGS-CRP | -0.005 | [-0.016, 0.005] | 0.294 | 0.440 |
| Putamen | PGS-CRP x Loss | 0.005 | [-0.004, 0.013] | 0.272 | 0.434 |
| Putamen | Scanner: Philips | -0.001 | [-0.026, 0.024] | 0.921 | 0.921 |
| Putamen | Scanner: Siemens | -0.009 | [-0.025, 0.008] | 0.301 | 0.451 |
| Putamen | Sex (Female) | -0.001 | [-0.010, 0.008] | 0.848 | 0.912 |

*Note.* All fixed-effect estimates from the cross-sectional Model 2, including all covariate terms. Body mass index (BMI) and principal genetic components (PC1-10) are scaled in the model. Abbreviations: ROI = region of interest; β = beta coefficient representing the difference in percent signal change between High and Low PGS-CRP groups; 95% CI = 95% confidence interval; *p*_FDR_ = Benjamini-Hochberg false discovery rate-corrected p-value; BMI = body mass index; PGS-CRP = polygenic score for C-reactive protein.

# Supplementary Table S7. Summary of Model 2 Longitudinal Sensitivity Analysis Effects

| **ROI** | **Term** | **β** | **95% CI** | ***p*** | ***p*_FDR_** |
| --- | --- | --- | --- | --- | --- |
| Caudate | PGS-CRP | -0.004 | [-0.012, 0.005] | 0.416 | 0.623 |
| Caudate | PGS-CRP x Loss | 0.005 | [-0.004, 0.014] | 0.314 | 0.471 |
| Nucleus Accumbens | PGS-CRP | 0.002 | [-0.009, 0.012] | 0.749 | 0.749 |
| Nucleus Accumbens | PGS-CRP x Loss | -0.003 | [-0.013, 0.007] | 0.531 | 0.531 |
| Putamen | PGS-CRP | -0.004 | [-0.011, 0.003] | 0.291 | 0.623 |
| Putamen | PGS-CRP x Loss | 0.005 | [-0.002, 0.012] | 0.189 | 0.471 |

*Note.* Fixed-effect estimates from longitudinal Model 2 across four imaging timepoints. Age was decomposed into baseline age and within-person age change from baseline. Abbreviations: ROI = region of interest; β = beta coefficient; CI = confidence interval; *p*_FDR_ = Benjamini–Hochberg false discovery rate-corrected p-value; PGS-CRP = polygenic score for C-reactive protein.

# Supplementary Table S8. PGS-CRP Effect Estimates for Striatal Activation During Reward and Loss Feedback (Cross-Sectional Model 3)

| **ROI** | **β** | **95% CI** | **p** | ***p*_FDR_** |
| --- | --- | --- | --- | --- |
| **PGS-CRP Main Effect (Reward Feedback)** | | | |  |
| Nucleus Accumbens | -0.002 | [-0.018, 0.013] | 0.765 | 0.765 |
| Caudate | 0.005 | [-0.010, 0.021] | 0.493 | 0.765 |
| Putamen | 0.003 | [-0.010, 0.016] | 0.656 | 0.765 |
| **PGS-CRP × Loss Feedback Interaction** | | | |  |
| Nucleus Accumbens | 0.012 | [-0.008, 0.032] | 0.247 | 0.247 |
| Caudate | 0.015 | [-0.007, 0.037] | 0.188 | 0.247 |
| Putamen | 0.010 | [-0.007, 0.027] | 0.242 | 0.247 |

*Note.* Main results for cross-sectional Model 3. PGS-CRP main effect estimates activation difference between PGS-CRP groups during reward feedback trials relative to loss feedback trials. PGS-CRP × loss feedback interaction estimates additional shift in this group difference for loss feedback trials relative to reward feedback trials. *Abbreviations:* ROI = region of interest; β = beta coefficient; CI = confidence interval; *p*_FDR_ = Benjamini-Hochberg false discovery rate-corrected p-value; PGS-CRP = polygenic score for C-reactive protein.

# Supplementary Table S9. Complete Fixed-Effect Output for Cross-Sectional Model 3

| **ROI** | **Term** | **β** | **95% CI** | ***p*** | ***p*_FDR_** |
| --- | --- | --- | --- | --- | --- |
| Caudate | Age at baseline | -0.003 | [-0.012, 0.006] | 0.527 | 0.790 |
| Caudate | BMI | 0.003 | [-0.003, 0.009] | 0.310 | 0.310 |
| Caudate | Caregiver edu: Bachelor's | -0.038 | [-0.130, 0.054] | 0.416 | 0.624 |
| Caudate | Caregiver edu: Graduate/professional | -0.039 | [-0.131, 0.054] | 0.412 | 0.618 |
| Caudate | Caregiver edu: HS/GED | -0.023 | [-0.119, 0.073] | 0.640 | 0.644 |
| Caudate | Caregiver edu: Some college | -0.024 | [-0.116, 0.068] | 0.613 | 0.613 |
| Caudate | Domain (loss) | -0.024 | [-0.039, -0.008] | 0.003 | 0.004 |
| Caudate | Income: 100k–200k | 0.010 | [-0.022, 0.042] | 0.541 | 0.812 |
| Caudate | Income: 25k–50k | 0.009 | [-0.026, 0.043] | 0.620 | 0.696 |
| Caudate | Income: 50k–75k | -0.003 | [-0.036, 0.031] | 0.880 | 0.880 |
| Caudate | Income: 75k–100k | -0.008 | [-0.041, 0.025] | 0.636 | 0.876 |
| Caudate | Income: >200k | 0.017 | [-0.018, 0.051] | 0.339 | 0.378 |
| Caudate | Intercept | 0.096 | [-0.032, 0.225] | 0.143 | 0.143 |
| Caudate | PC1 | -0.005 | [-0.012, 0.003] | 0.203 | 0.411 |
| Caudate | PC2 | 0.003 | [-0.003, 0.010] | 0.328 | 0.328 |
| Caudate | PC3 | -0.008 | [-0.014, -0.002] | 0.011 | 0.017 |
| Caudate | PC4 | -0.005 | [-0.014, 0.003] | 0.191 | 0.191 |
| Caudate | PC5 | 0.001 | [-0.008, 0.010] | 0.853 | 0.853 |
| Caudate | PC6 | 0.003 | [-0.004, 0.011] | 0.404 | 0.606 |
| Caudate | PC7 | -0.004 | [-0.011, 0.003] | 0.253 | 0.407 |
| Caudate | PC8 | 0.001 | [-0.008, 0.009] | 0.853 | 0.853 |
| Caudate | PC9 | 0.002 | [-0.004, 0.009] | 0.521 | 0.782 |
| Caudate | PC10 | -0.004 | [-0.013, 0.004] | 0.290 | 0.435 |
| Caudate | PGS-CRP | 0.005 | [-0.010, 0.021] | 0.493 | 0.765 |
| Caudate | PGS-CRP x Loss | 0.015 | [-0.007, 0.037] | 0.188 | 0.247 |
| Caudate | Scanner: Philips | 0.037 | [0.009, 0.066] | 0.016 | 0.048 |
| Caudate | Scanner: Siemens | 0.022 | [0.003, 0.041] | 0.029 | 0.029 |
| Caudate | Sex (Female) | -0.003 | [-0.014, 0.008] | 0.643 | 0.643 |
| Nucleus Accumbens | Age at baseline | -0.000 | [-0.010, 0.009] | 0.945 | 0.945 |
| Nucleus Accumbens | BMI | 0.005 | [-0.001, 0.011] | 0.094 | 0.282 |
| Nucleus Accumbens | Caregiver edu: Bachelor's | -0.073 | [-0.172, 0.026] | 0.148 | 0.443 |
| Nucleus Accumbens | Caregiver edu: Graduate/professional | -0.070 | [-0.169, 0.028] | 0.163 | 0.490 |
| Nucleus Accumbens | Caregiver edu: HS/GED | -0.068 | [-0.171, 0.035] | 0.197 | 0.592 |
| Nucleus Accumbens | Caregiver edu: Some college | -0.071 | [-0.169, 0.028] | 0.159 | 0.478 |
| Nucleus Accumbens | Domain (loss) | -0.047 | [-0.061, -0.033] | <.001 | <.001 |
| Nucleus Accumbens | Income: 100k–200k | 0.002 | [-0.033, 0.036] | 0.928 | 0.928 |
| Nucleus Accumbens | Income: 25k–50k | 0.007 | [-0.030, 0.044] | 0.696 | 0.696 |
| Nucleus Accumbens | Income: 50k–75k | 0.009 | [-0.026, 0.045] | 0.609 | 0.880 |
| Nucleus Accumbens | Income: 75k–100k | 0.003 | [-0.032, 0.039] | 0.848 | 0.876 |
| Nucleus Accumbens | Income: >200k | 0.017 | [-0.020, 0.054] | 0.378 | 0.378 |
| Nucleus Accumbens | Intercept | 0.200 | [0.061, 0.338] | 0.005 | 0.007 |
| Nucleus Accumbens | PC1 | -0.001 | [-0.010, 0.007] | 0.715 | 0.715 |
| Nucleus Accumbens | PC2 | 0.007 | [-0.001, 0.014] | 0.073 | 0.182 |
| Nucleus Accumbens | PC3 | -0.009 | [-0.016, -0.003] | 0.007 | 0.017 |
| Nucleus Accumbens | PC4 | -0.008 | [-0.017, 0.000] | 0.064 | 0.191 |
| Nucleus Accumbens | PC5 | -0.003 | [-0.013, 0.006] | 0.482 | 0.853 |
| Nucleus Accumbens | PC6 | 0.002 | [-0.006, 0.010] | 0.609 | 0.609 |
| Nucleus Accumbens | PC7 | -0.001 | [-0.008, 0.007] | 0.889 | 0.889 |
| Nucleus Accumbens | PC8 | 0.002 | [-0.007, 0.011] | 0.687 | 0.853 |
| Nucleus Accumbens | PC9 | 0.005 | [-0.002, 0.012] | 0.166 | 0.497 |
| Nucleus Accumbens | PC10 | -0.002 | [-0.011, 0.007] | 0.716 | 0.716 |
| Nucleus Accumbens | PGS-CRP | -0.002 | [-0.018, 0.013] | 0.765 | 0.765 |
| Nucleus Accumbens | PGS-CRP x Loss | 0.012 | [-0.008, 0.032] | 0.247 | 0.247 |
| Nucleus Accumbens | Scanner: Philips | 0.038 | [0.002, 0.073] | 0.048 | 0.048 |
| Nucleus Accumbens | Scanner: Siemens | 0.042 | [0.019, 0.065] | 0.001 | 0.004 |
| Nucleus Accumbens | Sex (Female) | -0.013 | [-0.025, -0.001] | 0.036 | 0.053 |
| Putamen | Age at baseline | -0.008 | [-0.016, -0.000] | 0.047 | 0.141 |
| Putamen | BMI | 0.003 | [-0.002, 0.008] | 0.224 | 0.310 |
| Putamen | Caregiver edu: Bachelor's | 0.011 | [-0.073, 0.096] | 0.789 | 0.789 |
| Putamen | Caregiver edu: Graduate/professional | 0.015 | [-0.069, 0.099] | 0.731 | 0.731 |
| Putamen | Caregiver edu: HS/GED | 0.021 | [-0.067, 0.108] | 0.644 | 0.644 |
| Putamen | Caregiver edu: Some college | 0.027 | [-0.057, 0.111] | 0.527 | 0.613 |
| Putamen | Domain (loss) | 0.014 | [0.002, 0.026] | 0.019 | 0.019 |
| Putamen | Income: 100k–200k | 0.016 | [-0.013, 0.046] | 0.272 | 0.812 |
| Putamen | Income: 25k–50k | 0.021 | [-0.011, 0.052] | 0.194 | 0.583 |
| Putamen | Income: 50k–75k | 0.005 | [-0.025, 0.035] | 0.735 | 0.880 |
| Putamen | Income: 75k–100k | 0.002 | [-0.028, 0.033] | 0.876 | 0.876 |
| Putamen | Income: >200k | 0.021 | [-0.010, 0.053] | 0.187 | 0.378 |
| Putamen | Intercept | 0.181 | [0.063, 0.298] | 0.003 | 0.007 |
| Putamen | PC1 | -0.004 | [-0.011, 0.003] | 0.274 | 0.411 |
| Putamen | PC2 | 0.005 | [-0.001, 0.011] | 0.122 | 0.182 |
| Putamen | PC3 | -0.004 | [-0.010, 0.001] | 0.131 | 0.131 |
| Putamen | PC4 | -0.005 | [-0.013, 0.002] | 0.176 | 0.191 |
| Putamen | PC5 | -0.002 | [-0.010, 0.006] | 0.704 | 0.853 |
| Putamen | PC6 | 0.003 | [-0.004, 0.010] | 0.369 | 0.606 |
| Putamen | PC7 | -0.004 | [-0.010, 0.003] | 0.271 | 0.407 |
| Putamen | PC8 | 0.002 | [-0.006, 0.010] | 0.561 | 0.853 |
| Putamen | PC9 | -0.001 | [-0.007, 0.005] | 0.826 | 0.826 |
| Putamen | PC10 | -0.006 | [-0.013, 0.002] | 0.155 | 0.435 |
| Putamen | PGS-CRP | 0.003 | [-0.010, 0.016] | 0.656 | 0.765 |
| Putamen | PGS-CRP x Loss | 0.010 | [-0.007, 0.027] | 0.242 | 0.247 |
| Putamen | Scanner: Philips | 0.030 | [0.003, 0.057] | 0.042 | 0.048 |
| Putamen | Scanner: Siemens | 0.030 | [0.012, 0.047] | 0.003 | 0.005 |
| Putamen | Sex (Female) | -0.014 | [-0.024, -0.004] | 0.007 | 0.020 |

*Note.* All fixed-effect estimates from the cross-sectional Model 3, including all covariate terms. Abbreviations: ROI = region of interest; β = beta coefficient; CI = confidence interval; *p*_FDR_ = Benjamini–Hochberg false discovery rate-corrected p-value; PGS-CRP = polygenic score for C-reactive protein.

# Supplementary Table S10. Summary of Model 3 Longitudinal Sensitivity Analysis Effects

| **ROI** | **Term** | **β** | **95% CI** | ***p*** | ***p*_FDR_** |
| --- | --- | --- | --- | --- | --- |
| Caudate | Domain (loss) | -0.023 | [-0.031, -0.014] | <.001 | <.001 |
| Caudate | Intercept | 0.109 | [0.026, 0.192] | 0.010 | 0.010 |
| Caudate | PGS-CRP | 0.001 | [-0.009, 0.011] | 0.826 | 0.826 |
| Caudate | PGS-CRP x Loss | 0.010 | [-0.002, 0.023] | 0.107 | 0.267 |
| Nucleus Accumbens | Domain (loss) | -0.038 | [-0.048, -0.028] | <.001 | <.001 |
| Nucleus Accumbens | Intercept | 0.241 | [0.144, 0.339] | <.001 | <.001 |
| Nucleus Accumbens | PGS-CRP | 0.002 | [-0.009, 0.013] | 0.701 | 0.826 |
| Nucleus Accumbens | PGS-CRP x Loss | 0.003 | [-0.011, 0.017] | 0.652 | 0.652 |
| Putamen | Domain (loss) | 0.015 | [0.008, 0.023] | <.001 | <.001 |
| Putamen | Intercept | 0.190 | [0.119, 0.262] | <.001 | <.001 |
| Putamen | PGS-CRP | 0.002 | [-0.006, 0.010] | 0.649 | 0.826 |
| Putamen | PGS-CRP x Loss | 0.007 | [-0.003, 0.018] | 0.178 | 0.267 |

*Note.* Fixed-effect estimates from longitudinal Model 3. Age was decomposed into baseline age and within-person age change from baseline. Abbreviations: ROI = region of interest; β = beta coefficient; CI = confidence interval; *p*_FDR_ = Benjamini–Hochberg false discovery rate-corrected p-value; PGS-CRP = polygenic score for C-reactive protein.

# Supplementary Table S11. Summary of Cross-Sectional Model 1 Clinical Covariate Sensitivity Analysis Effects

| **ROI** | **Term** | **β** | **95% CI** | ***p*** | ***p*_FDR_** |
| --- | --- | --- | --- | --- | --- |
| Caudate | PGS-CRP | 0.001 | [-0.013, 0.015] | 0.864 | 0.864 |
| Caudate | PGS-CRP x Large Loss | -0.009 | [-0.022, 0.005] | 0.223 | 0.381 |
| Caudate | PGS-CRP x Large Win | -0.017 | [-0.031, -0.004] | 0.013 | 0.022 |
| Caudate | PGS-CRP x Small Loss | 0.002 | [-0.012, 0.016] | 0.779 | 0.779 |
| Nucleus Accumbens | PGS-CRP | 0.007 | [-0.008, 0.022] | 0.356 | 0.864 |
| Nucleus Accumbens | PGS-CRP x Large Loss | -0.007 | [-0.021, 0.008] | 0.381 | 0.381 |
| Nucleus Accumbens | PGS-CRP x Large Win | -0.017 | [-0.032, -0.002] | 0.022 | 0.022 |
| Nucleus Accumbens | PGS-CRP x Small Loss | -0.004 | [-0.018, 0.011] | 0.610 | 0.779 |
| Putamen | PGS-CRP | 0.001 | [-0.011, 0.013] | 0.855 | 0.864 |
| Putamen | PGS-CRP x Large Loss | -0.006 | [-0.017, 0.006] | 0.334 | 0.381 |
| Putamen | PGS-CRP x Large Win | -0.014 | [-0.025, -0.002] | 0.021 | 0.022 |
| Putamen | PGS-CRP x Small Loss | 0.002 | [-0.010, 0.013] | 0.743 | 0.779 |

*Note.* Fixed-effect estimates for PGS-CRP-related terms from the cross-sectional Model 1 sensitivity analysis, which additionally adjusted for psychiatric comorbidity and psychotropic medication use at baseline. Model structure otherwise mirrored the primary cross-sectional Model 1. Abbreviations: ROI = region of interest; β = beta coefficient; CI = confidence interval; *p*_FDR_ = Benjamini-Hochberg false discovery rate-corrected p-value; PGS-CRP = polygenic score for C-reactive protein.

# Supplementary Table S12. Summary of Cross-Sectional Model 2 Clinical Covariate Sensitivity Analysis Effects

| **ROI** | **Term** | **β** | **95% CI** | ***p*** | ***p*_FDR_** |
| --- | --- | --- | --- | --- | --- |
| Caudate | PGS-CRP | -0.007 | [-0.020, 0.005] | 0.235 | 0.449 |
| Caudate | PGS-CRP x Loss | 0.005 | [-0.005, 0.016] | 0.290 | 0.435 |
| Nucleus Accumbens | PGS-CRP | -0.001 | [-0.014, 0.012] | 0.875 | 0.875 |
| Nucleus Accumbens | PGS-CRP x Loss | 0.003 | [-0.008, 0.014] | 0.592 | 0.592 |
| Putamen | PGS-CRP | -0.005 | [-0.016, 0.005] | 0.300 | 0.449 |
| Putamen | PGS-CRP x Loss | 0.005 | [-0.004, 0.013] | 0.272 | 0.435 |

*Note.* Fixed-effect estimates for PGS-CRP-related terms from the cross-sectional Model 2 sensitivity analysis, which additionally adjusted for psychiatric comorbidity and psychotropic medication use at baseline. Model structure otherwise mirrored the primary cross-sectional Model 2. Abbreviations: ROI = region of interest; β = beta coefficient; CI = confidence interval; *p*_FDR_ = Benjamini-Hochberg false discovery rate-corrected p-value; PGS-CRP = polygenic score for C-reactive protein.

# Supplementary Table S13. Summary of Cross-Sectional Model 3 Clinical Covariate Sensitivity Analysis Effects

| **ROI** | **Term** | **β** | **95% CI** | ***p*** | ***p*_FDR_** |
| --- | --- | --- | --- | --- | --- |
| Caudate | PGS-CRP | 0.006 | [-0.010, 0.021] | 0.470 | 0.795 |
| Caudate | PGS-CRP x Loss | 0.015 | [-0.007, 0.037] | 0.188 | 0.247 |
| Nucleus Accumbens | PGS-CRP | -0.002 | [-0.018, 0.014] | 0.795 | 0.795 |
| Nucleus Accumbens | PGS-CRP x Loss | 0.012 | [-0.008, 0.032] | 0.247 | 0.247 |
| Putamen | PGS-CRP | 0.003 | [-0.010, 0.017] | 0.609 | 0.795 |
| Putamen | PGS-CRP x Loss | 0.010 | [-0.007, 0.027] | 0.242 | 0.247 |

*Note.* Fixed-effect estimates for PGS-CRP-related terms from the cross-sectional Model 3 sensitivity analysis, which additionally adjusted for psychiatric comorbidity and psychotropic medication use at baseline. Model structure otherwise mirrored the primary cross-sectional Model 3. Abbreviations: ROI = region of interest; β = beta coefficient; CI = confidence interval; *p*_FDR_ = Benjamini-Hochberg false discovery rate-corrected p-value; PGS-CRP = polygenic score for C-reactive protein.

# Supplementary Table S14. Summary of Longitudinal Model 1 Clinical Covariate Sensitivity Analysis Effects

| **ROI** | **Term** | **β** | **95% CI** | ***p*** | ***p*_FDR_** |
| --- | --- | --- | --- | --- | --- |
| Caudate | PGS-CRP | 0.002 | [-0.008, 0.013] | 0.640 | 0.823 |
| Caudate | PGS-CRP x Large Loss | -0.002 | [-0.013, 0.008] | 0.698 | 0.938 |
| Caudate | PGS-CRP x Large Win | -0.013 | [-0.023, -0.002] | 0.016 | 0.048 |
| Caudate | PGS-CRP x Small Loss | 0.000 | [-0.010, 0.011] | 0.938 | 0.938 |
| Nucleus Accumbens | PGS-CRP | 0.007 | [-0.004, 0.019] | 0.223 | 0.668 |
| Nucleus Accumbens | PGS-CRP x Large Loss | -0.010 | [-0.022, 0.002] | 0.098 | 0.295 |
| Nucleus Accumbens | PGS-CRP x Large Win | -0.010 | [-0.022, 0.002] | 0.091 | 0.091 |
| Nucleus Accumbens | PGS-CRP x Small Loss | -0.007 | [-0.018, 0.005] | 0.270 | 0.517 |
| Putamen | PGS-CRP | -0.001 | [-0.009, 0.007] | 0.823 | 0.823 |
| Putamen | PGS-CRP x Large Loss | -0.000 | [-0.009, 0.008] | 0.938 | 0.938 |
| Putamen | PGS-CRP x Large Win | -0.007 | [-0.016, 0.001] | 0.091 | 0.091 |
| Putamen | PGS-CRP x Small Loss | 0.004 | [-0.004, 0.013] | 0.345 | 0.517 |

*Note.* Fixed-effect estimates for PGS-CRP-related terms from the longitudinal Model 1 sensitivity analysis, which additionally adjusted for time-varying ADHD and PTSD diagnoses and current psychotropic medication use. Oppositional defiant disorder and other mood disorders were excluded because the corresponding KSADS-COMP items were not administered consistently across visits. Model structure otherwise mirrored the primary longitudinal Model 1. Abbreviations: ROI = region of interest; β = beta coefficient; CI = confidence interval; *p*_FDR_ = Benjamini-Hochberg false discovery rate-corrected p-value; PGS-CRP = polygenic score for C-reactive protein; ADHD = attention-deficit/hyperactivity disorder; PTSD = post-traumatic stress disorder.

# Supplementary Table S15. Summary of Longitudinal Model 2 Clinical Covariate Sensitivity Analysis Effects

| **ROI** | **Term** | **β** | **95% CI** | ***p*** | ***p*_FDR_** |
| --- | --- | --- | --- | --- | --- |
| Caudate | PGS-CRP | -0.004 | [-0.013, 0.005] | 0.344 | 0.516 |
| Caudate | PGS-CRP x Loss | 0.006 | [-0.003, 0.015] | 0.230 | 0.346 |
| Nucleus Accumbens | PGS-CRP | 0.003 | [-0.008, 0.013] | 0.618 | 0.618 |
| Nucleus Accumbens | PGS-CRP x Loss | -0.003 | [-0.014, 0.007] | 0.508 | 0.508 |
| Putamen | PGS-CRP | -0.004 | [-0.012, 0.003] | 0.247 | 0.516 |
| Putamen | PGS-CRP x Loss | 0.005 | [-0.002, 0.013] | 0.151 | 0.346 |

*Note.* Fixed-effect estimates for PGS-CRP-related terms from the longitudinal Model 2 sensitivity analysis, which additionally adjusted for time-varying ADHD and PTSD diagnoses and current psychotropic medication use. Oppositional defiant disorder and other mood disorders were excluded because the corresponding KSADS-COMP items were not administered consistently across visits. Model structure otherwise mirrored the primary longitudinal Model 2. Abbreviations: ROI = region of interest; β = beta coefficient; CI = confidence interval; *p*_FDR_ = Benjamini-Hochberg false discovery rate-corrected p-value; PGS-CRP = polygenic score for C-reactive protein; ADHD = attention-deficit/hyperactivity disorder; PTSD = post-traumatic stress disorder.

# Supplementary Table S16. Summary of Longitudinal Model 3 Clinical Covariate Sensitivity Analysis Effects

| **ROI** | **Term** | **β** | **95% CI** | ***p*** | ***p*_FDR_** |
| --- | --- | --- | --- | --- | --- |
| Caudate | PGS-CRP | 0.002 | [-0.008, 0.012] | 0.707 | 0.707 |
| Caudate | PGS-CRP x Loss | 0.010 | [-0.003, 0.022] | 0.143 | 0.256 |
| Nucleus Accumbens | PGS-CRP | 0.003 | [-0.008, 0.014] | 0.619 | 0.707 |
| Nucleus Accumbens | PGS-CRP x Loss | 0.002 | [-0.012, 0.016] | 0.751 | 0.751 |
| Putamen | PGS-CRP | 0.002 | [-0.007, 0.010] | 0.666 | 0.707 |
| Putamen | PGS-CRP x Loss | 0.007 | [-0.003, 0.018] | 0.171 | 0.256 |

*Note.* Fixed-effect estimates for PGS-CRP-related terms from the longitudinal Model 3 sensitivity analysis, which additionally adjusted for time-varying ADHD and PTSD diagnoses and current psychotropic medication use. Oppositional defiant disorder and other mood disorders were excluded because the corresponding KSADS-COMP items were not administered consistently across visits. Model structure otherwise mirrored the primary longitudinal Model 3. Abbreviations: ROI = region of interest; β = beta coefficient; CI = confidence interval; *p*_FDR_ = Benjamini-Hochberg false discovery rate-corrected p-value; PGS-CRP = polygenic score for C-reactive protein; ADHD = attention-deficit/hyperactivity disorder; PTSD = post-traumatic stress disorder.

# Supplementary Table S17. Summary of Continuous PGS-CRP Sensitivity Analysis Effects (Model 1)

| **ROI** | **β** | **95% CI** | ***p*** | ***p*_FDR_** |
| --- | --- | --- | --- | --- |
| **Cross-Sectional: PGS-CRP Main Effect on Small Reward Anticipation** | | | |  |
| Nucleus Accumbens | 0.005 | [-0.003, 0.012] | 0.219 | 0.656 |
| Caudate | 0.001 | [-0.006, 0.008] | 0.780 | 0.780 |
| Putamen | 0.001 | [-0.005, 0.007] | 0.681 | 0.780 |
| **Cross-Sectional: PGS-CRP x Large Reward Anticipation Interaction** | | | |  |
| Nucleus Accumbens | -0.006 | [-0.014, 0.001] | 0.082 | 0.247 |
| Caudate | -0.003 | [-0.010, 0.004] | 0.380 | 0.380 |
| Putamen | -0.003 | [-0.009, 0.002] | 0.246 | 0.369 |
| **Cross-Sectional: PGS-CRP x Small Loss Anticipation Interaction** | | | |  |
| Nucleus Accumbens | -0.003 | [-0.010, 0.005] | 0.491 | 0.580 |
| Caudate | 0.002 | [-0.005, 0.009] | 0.548 | 0.580 |
| Putamen | 0.002 | [-0.004, 0.007] | 0.580 | 0.580 |
| **Cross-Sectional: PGS-CRP x Large Loss Anticipation Interaction** | | | |  |
| Nucleus Accumbens | -0.002 | [-0.009, 0.005] | 0.561 | 0.842 |
| Caudate | -0.000 | [-0.007, 0.007] | 0.961 | 0.961 |
| Putamen | -0.002 | [-0.008, 0.004] | 0.556 | 0.842 |
| **Longitudinal: PGS-CRP Main Effect on Small Reward Anticipation** | | | |  |
| Nucleus Accumbens | 0.004 | [-0.002, 0.010] | 0.200 | 0.425 |
| Caudate | 0.003 | [-0.002, 0.008] | 0.284 | 0.425 |
| Putamen | -0.000 | [-0.004, 0.004] | 0.997 | 0.997 |
| **Longitudinal: PGS-CRP x Large Reward Anticipation Interaction** | | | |  |
| Nucleus Accumbens | -0.005 | [-0.011, 0.001] | 0.115 | 0.172 |
| Caudate | -0.004 | [-0.009, 0.001] | 0.109 | 0.172 |
| Putamen | -0.002 | [-0.006, 0.002] | 0.312 | 0.312 |
| **Longitudinal: PGS-CRP x Small Loss Anticipation Interaction** | | | |  |
| Nucleus Accumbens | -0.002 | [-0.008, 0.004] | 0.513 | 0.769 |
| Caudate | -0.000 | [-0.005, 0.005] | 0.968 | 0.968 |
| Putamen | 0.002 | [-0.002, 0.006] | 0.364 | 0.769 |
| **Longitudinal: PGS-CRP x Large Loss Anticipation Interaction** | | | |  |
| Nucleus Accumbens | -0.002 | [-0.008, 0.004] | 0.512 | 0.955 |
| Caudate | 0.000 | [-0.005, 0.005] | 0.955 | 0.955 |
| Putamen | 0.000 | [-0.004, 0.005] | 0.892 | 0.955 |

*Note.* Fixed-effect estimates for PGS-CRP-related terms from Model 1 refit with PGS-CRP entered as a continuous, z-scored predictor, presented for the cross-sectional and longitudinal samples. Estimates reflect the change in activation per one standard deviation increase in PGS-CRP. Model structure otherwise mirrored the primary analysis. *Abbreviations:* ROI = region of interest; β = beta coefficient; CI = confidence interval; *p*_FDR_ = Benjamini-Hochberg false discovery rate-corrected p-value; PGS-CRP = polygenic score for C-reactive protein.

# Supplementary Table S18. Summary of Quintile and Quadratic PGS-CRP Sensitivity Analysis Effects for Large Reward Anticipation (Model 1)

| **ROI** | **β** | **95% CI** | ***p*** | ***p*_FDR_** |
| --- | --- | --- | --- | --- |
| **Cross-Sectional: Q2 vs. Q1 x Large Reward Anticipation Interaction** | | | |  |
| Nucleus Accumbens | -0.009 | [-0.032, 0.014] | 0.425 | 0.886 |
| Caudate | 0.003 | [-0.019, 0.025] | 0.771 | 0.886 |
| Putamen | 0.001 | [-0.017, 0.020] | 0.886 | 0.886 |
| **Cross-Sectional: Q3 vs. Q1 x Large Reward Anticipation Interaction** | | | |  |
| Nucleus Accumbens | -0.017 | [-0.040, 0.006] | 0.152 | 0.248 |
| Caudate | -0.015 | [-0.037, 0.007] | 0.175 | 0.248 |
| Putamen | -0.011 | [-0.029, 0.007] | 0.248 | 0.248 |
| **Cross-Sectional: Q4 vs. Q1 x Large Reward Anticipation Interaction** | | | |  |
| Nucleus Accumbens | -0.027 | [-0.050, -0.004] | 0.022 | 0.035* |
| Caudate | -0.025 | [-0.047, -0.003] | 0.023 | 0.035* |
| Putamen | -0.019 | [-0.037, -0.001] | 0.044 | 0.044* |
| **Cross-Sectional: Q5 vs. Q1 x Large Reward Anticipation Interaction** | | | |  |
| Nucleus Accumbens | -0.019 | [-0.042, 0.004] | 0.101 | 0.303 |
| Caudate | -0.002 | [-0.024, 0.020] | 0.836 | 0.836 |
| Putamen | -0.003 | [-0.021, 0.016] | 0.768 | 0.836 |
| **Cross-Sectional: Quadratic PGS-CRP x Large Reward Anticipation Interaction** |  |  |  |  |
| Nucleus Accumbens | -0.130 | [-1.145, 0.885] | 0.802 | 0.802 |
| Caudate | -0.288 | [-1.248, 0.672] | 0.557 | 0.802 |
| Putamen | -0.301 | [-1.103, 0.500] | 0.461 | 0.802 |
| **Longitudinal: Q2 vs. Q1 x Large Reward Anticipation Interaction** | | | |  |
| Nucleus Accumbens | -0.009 | [-0.028, 0.009] | 0.323 | 0.747 |
| Caudate | 0.006 | [-0.011, 0.022] | 0.498 | 0.747 |
| Putamen | 0.002 | [-0.012, 0.015] | 0.819 | 0.819 |
| **Longitudinal: Q3 vs. Q1 x Large Reward Anticipation Interaction** | | | |  |
| Nucleus Accumbens | -0.018 | [-0.036, 0.000] | 0.056 | 0.136 |
| Caudate | -0.014 | [-0.030, 0.002] | 0.091 | 0.136 |
| Putamen | -0.008 | [-0.021, 0.006] | 0.253 | 0.253 |
| **Longitudinal: Q4 vs. Q1 x Large Reward Anticipation Interaction** | | | |  |
| Nucleus Accumbens | -0.019 | [-0.038, -0.001] | 0.039 | 0.059 |
| Caudate | -0.018 | [-0.034, -0.001] | 0.034 | 0.059 |
| Putamen | -0.013 | [-0.026, 0.001] | 0.062 | 0.062 |
| **Longitudinal: Q5 vs. Q1 x Large Reward Anticipation Interaction** | | | |  |
| Nucleus Accumbens | -0.013 | [-0.031, 0.005] | 0.167 | 0.502 |
| Caudate | -0.003 | [-0.019, 0.013] | 0.700 | 0.848 |
| Putamen | 0.001 | [-0.012, 0.015] | 0.848 | 0.848 |
| **Longitudinal: Quadratic PGS-CRP x Large Reward Anticipation Interaction** |  |  |  |  |
| Nucleus Accumbens | 0.343 | [-0.924, 1.610] | 0.596 | 0.917 |
| Caudate | 0.059 | [-1.060, 1.178] | 0.917 | 0.917 |
| Putamen | 0.238 | [-0.686, 1.161] | 0.614 | 0.917 |

*Note*. Fixed-effect estimates characterizing the shape of the PGS-CRP association across its distribution, presented for the cross-sectional and longitudinal samples. Quintile estimates are the PGS-CRP quintile by large reward anticipation interaction from Model 1 refit with PGS-CRP entered as quintiles of the sample distribution, with the lowest quintile as the reference group. Quadratic estimates are the quadratic PGS-CRP by large reward anticipation interaction from Model 1 refit with PGS-CRP entered as a continuous predictor with a quadratic term, fitted using orthogonal polynomials; a significant quadratic term would indicate curvature in the association across the distribution. Estimates for the quadratic term are on the orthogonal polynomial scale and are therefore not comparable in magnitude to the quintile contrasts. Model structure otherwise mirrored the primary analysis. *Abbreviations:* ROI = region of interest; β = beta coefficient; CI = confidence interval; *p*_FDR_ = Benjamini-Hochberg false discovery rate-corrected p-value; PGS-CRP = polygenic score for C-reactive protein; Q1–Q5 = first through fifth quintile of the PGS-CRP distribution.

# Supplementary Figure S3. PGS-CRP × Large Reward Anticipation Interaction Estimates by PGS-CRP Quintile

*Note.* Point estimates and 95% confidence intervals for the PGS-CRP quintile × large reward anticipation interaction from Model 1, refit with PGS-CRP entered as quintiles of the sample distribution. Estimates are expressed relative to the lowest quintile, such that negative values indicate attenuated differentiation between large and small reward anticipation relative to Q1. Columns show the three striatal regions and rows show the cross-sectional (baseline) and longitudinal samples; the dashed line marks the null. Significance markers reflect Benjamini-Hochberg false discovery rate correction applied across the three regions within each term: + p < .10, * p < .05, ** p < .01, *** p < .001. *Abbreviations:* CI = confidence interval; FDR = false discovery rate; PGS-CRP = polygenic score for C-reactive protein; Q1–Q5 = first through fifth quintile of the PGS-CRP distribution.

# Supplementary Table S19. Parent-Reported Pubertal Development Scale Scores by Imaging Visit and Sex

|  | **Year 0** | | **Year 2** | | **Year 4** | | **Year 6** | |
| --- | --- | --- | --- | --- | --- | --- | --- | --- |
| **Characteristic** | **Male** N = 2489 | **Female** N = 2313 | **Male** N = 1663 | **Female** N = 1436 | **Male** N = 1286 | **Female** N = 1126 | **Male** N = 905 | **Female** N = 842 |
| PDS mean score, M (SD) | 1.36 (0.30) | 1.65 (0.46) | 1.71 (0.53) | 2.39 (0.67) | 2.59 (0.57) | 3.29 (0.44) | 3.08 (0.39) | 3.68 (0.31) |
| Range | 1.0–3.0 | 1.0–3.4 | 1.0–3.2 | 1.0–4.0 | 1.0–4.0 | 1.4–4.0 | 1.2–4.0 | 2.2–4.0 |
| Stage classification available, n | 2374 | 2292 | 1503 | 1371 | 1219 | 1076 | 865 | 797 |
| **Pubertal stage, n (%)** |  |  |  |  |  |  |  |  |
| Prepubertal (stage 1) | 1947 (82.0%) | 883 (38.5%) | 668 (44.4%) | 80 (5.8%) | 67 (5.5%) | 1 (0.1%) | 1 (0.1%) | 0 (0.0%) |
| Early pubertal (stage 2) | 392 (16.5%) | 608 (26.5%) | 515 (34.3%) | 160 (11.7%) | 150 (12.3%) | 3 (0.3%) | 10 (1.2%) | 0 (0.0%) |
| Mid-pubertal (stage 3) | 32 (1.3%) | 774 (33.8%) | 260 (17.3%) | 706 (51.5%) | 483 (39.6%) | 100 (9.3%) | 168 (19.4%) | 6 (0.8%) |
| Late pubertal (stage 4) | 3 (0.1%) | 27 (1.2%) | 60 (4.0%) | 406 (29.6%) | 514 (42.2%) | 789 (73.3%) | 638 (73.8%) | 378 (47.4%) |
| Post-pubertal (stage 5) | 0 (0.0%) | 0 (0.0%) | 0 (0.0%) | 19 (1.4%) | 5 (0.4%) | 183 (17.0%) | 48 (5.5%) | 413 (51.8%) |

*Note.* Parent-reported Pubertal Development Scale (PDS) scores for participants contributing data at each imaging visit, presented by sex. The PDS mean score is the average of the five sex-specific items and ranges from 1 to 4, with higher scores indicating more advanced maturation. Pubertal stage is the approximate Tanner stage classification derived from the same items, which requires complete item data and is therefore available for fewer participants than the mean score; stage percentages are calculated among participants with an available classification. The PDS indexes parent-perceived rather than clinically assessed pubertal development. Abbreviations: PDS = Pubertal Development Scale; M = mean; SD = standard deviation; n = frequency.

# Supplementary Figure S4. Parent-Reported Pubertal Development Scale Stage by Imaging Visit and Sex

*Note.* Proportion of participants at each approximate Tanner stage derived from the parent-reported Pubertal Development Scale (PDS), shown at each imaging visit and separately by sex. Stages correspond to 1 = prepubertal, 2 = early pubertal, 3 = mid-pubertal, 4 = late pubertal, and 5 = post-pubertal. Stage classification requires complete PDS item data and is therefore available for fewer participants than the PDS mean score; proportions are calculated among participants with an available classification at each visit (see Supplementary Table S19). The PDS indexes parent-perceived rather than clinically assessed pubertal development. *Abbreviations:* PDS = Pubertal Development Scale.

# Supplementary Table S20. Summary of Longitudinal Model 1 Adjusted for Time-Varying Pubertal Status

| **ROI** | **β** | **95% CI** | ***p*** | ***p*_FDR_** |
| --- | --- | --- | --- | --- |
| **PGS-CRP Main Effect on Small Reward Anticipation** | | | |  |
| Nucleus Accumbens | 0.007 | [-0.005, 0.018] | 0.257 | 0.688 |
| Caudate | 0.004 | [-0.006, 0.014] | 0.459 | 0.688 |
| Putamen | -0.000 | [-0.008, 0.008] | 0.987 | 0.987 |
| **PGS-CRP x Large Reward Anticipation Interaction** | | | |  |
| Nucleus Accumbens | -0.010 | [-0.022, 0.002] | 0.090 | 0.090 |
| Caudate | -0.013 | [-0.024, -0.003] | 0.012 | 0.036 |
| Putamen | -0.008 | [-0.016, 0.001] | 0.084 | 0.090 |
| **PGS-CRP x Small Loss Anticipation Interaction** | | | |  |
| Nucleus Accumbens | -0.006 | [-0.018, 0.005] | 0.292 | 0.600 |
| Caudate | -0.001 | [-0.011, 0.010] | 0.893 | 0.893 |
| Putamen | 0.004 | [-0.005, 0.012] | 0.400 | 0.600 |
| **PGS-CRP x Large Loss Anticipation Interaction** | | | |  |
| Nucleus Accumbens | -0.010 | [-0.022, 0.002] | 0.098 | 0.294 |
| Caudate | -0.003 | [-0.014, 0.007] | 0.547 | 0.820 |
| Putamen | -0.001 | [-0.009, 0.008] | 0.823 | 0.823 |

*Note.* Fixed-effect estimates for PGS-CRP-related terms from the longitudinal Model 1 refit with parent-reported pubertal status included as a time-varying fixed-effect covariate. Analyses were restricted to observations with available pubertal status data. Model structure otherwise mirrored the primary longitudinal Model 1. Abbreviations: ROI = region of interest; β = beta coefficient; CI = confidence interval; *p*_FDR_ = Benjamini-Hochberg false discovery rate-corrected p-value; PGS-CRP = polygenic score for C-reactive protein.

# Supplementary Table S21. Summary of Pubertal Status Moderation of Longitudinal Model 1

| **ROI** | **β** | **95% CI** | **p** | ***p*_FDR_** |
| --- | --- | --- | --- | --- |
| **PGS-CRP Main Effect on Small Reward Anticipation** | | | |  |
| Nucleus Accumbens | 0.006 | [-0.005, 0.018] | 0.283 | 0.685 |
| Caudate | 0.004 | [-0.006, 0.014] | 0.457 | 0.685 |
| Putamen | 0.000 | [-0.008, 0.008] | 1.000 | 1.000 |
| **PGS-CRP x Large Reward Anticipation Interaction** | | | |  |
| Nucleus Accumbens | -0.010 | [-0.022, 0.002] | 0.090 | 0.090 |
| Caudate | -0.013 | [-0.023, -0.003] | 0.013 | 0.038 |
| Putamen | -0.007 | [-0.016, 0.001] | 0.088 | 0.090 |
| **PGS-CRP x Small Loss Anticipation Interaction** | | | |  |
| Nucleus Accumbens | -0.006 | [-0.018, 0.006] | 0.303 | 0.595 |
| Caudate | -0.001 | [-0.011, 0.010] | 0.904 | 0.904 |
| Putamen | 0.004 | [-0.005, 0.012] | 0.397 | 0.595 |
| **PGS-CRP x Large Loss Anticipation Interaction** | | | |  |
| Nucleus Accumbens | -0.010 | [-0.021, 0.002] | 0.104 | 0.313 |
| Caudate | -0.003 | [-0.013, 0.007] | 0.569 | 0.844 |
| Putamen | -0.001 | [-0.009, 0.008] | 0.844 | 0.844 |
| **PGS-CRP x Pubertal Status Interaction** | | | |  |
| Nucleus Accumbens | -0.004 | [-0.013, 0.005] | 0.413 | 0.883 |
| Caudate | -0.001 | [-0.008, 0.007] | 0.883 | 0.883 |
| Putamen | 0.001 | [-0.005, 0.008] | 0.654 | 0.883 |
| **PGS-CRP x Large Reward Anticipation x Pubertal Status Interaction** | | | |  |
| Nucleus Accumbens | 0.002 | [-0.010, 0.014] | 0.733 | 0.733 |
| Caudate | 0.004 | [-0.006, 0.015] | 0.407 | 0.733 |
| Putamen | 0.003 | [-0.006, 0.011] | 0.539 | 0.733 |
| **PGS-CRP x Small Loss Anticipation x Pubertal Status Interaction** | | | |  |
| Nucleus Accumbens | 0.000 | [-0.012, 0.011] | 0.967 | 0.967 |
| Caudate | 0.001 | [-0.010, 0.011] | 0.892 | 0.967 |
| Putamen | 0.000 | [-0.009, 0.008] | 0.922 | 0.967 |
| **PGS-CRP x Large Loss Anticipation x Pubertal Status Interaction** | | | |  |
| Nucleus Accumbens | -0.002 | [-0.014, 0.010] | 0.728 | 0.953 |
| Caudate | 0.004 | [-0.006, 0.015] | 0.415 | 0.953 |
| Putamen | 0.000 | [-0.009, 0.008] | 0.953 | 0.953 |

*Note.* Fixed-effect estimates from the longitudinal Model 1 refit with a three-way PGS-CRP x condition x pubertal status interaction. Pubertal status was the z-scored parent-reported Pubertal Development Scale mean score, entered as a time-varying predictor. Model structure otherwise mirrored the primary longitudinal Model 1. Abbreviations: ROI = region of interest; β = beta coefficient; CI = confidence interval; *p*_FDR_ = Benjamini-Hochberg false discovery rate-corrected p-value; PGS-CRP = polygenic score for C-reactive protein.

1. Low PGS-CRP
2. High PGS-CRP
3. BD includes bipolar I and II diagnoses
4. Other mood disorders include specified depressive disorders other than MDD, PDD, and DMDD, and specified and unspecified bipolar disorders other than bipolar I and II (i.e., subthreshold or minimum duration).
5. Low PGS-CRP (median split)
6. High PGS-CRP (median split)
7. Other externalizing disorders include unspecified ADHD and other specified ADHD (i.e., subthreshold or not otherwise specified).
8. Other anxiety disorders include subthreshold or other specified variants of GAD, OCD, social anxiety disorder, separation anxiety disorder, agoraphobia, and panic disorder.
9. Other ASD-related disorders include other specified neurodevelopmental disorder (i.e., ASD subthreshold).
10. Other mood disorders include specified depressive disorders other than MDD, PDD, and DMDD, and specified and unspecified bipolar disorders other than bipolar I and II (i.e., subthreshold or minimum duration).
11. Other psychotic disorders include unspecified schizophrenia spectrum and other psychotic disorders, other specified psychotic disorder (i.e., subthreshold), and attenuated psychotic symptoms including hallucinations, delusions, and associated psychotic symptoms.
12. Other stress-related disorders include other specified trauma- and stressor-related disorder (i.e., PTSD subthreshold).
13. Other tic disorders include unspecified tic disorder.
14. Low PGS-CRP (median split)
15. High PGS-CRP (median split)
